# Assessment of Sexual and Reproductive Health Status of Street Children in Addis Ababa

**DOI:** 10.1155/2013/524076

**Published:** 2013-12-26

**Authors:** Demelash Habtamu, Addisie Adamu

**Affiliations:** ^1^College of Medicine and Health Science, Madawalabu University, Bale, Goba, Ethiopia; ^2^College of Health Science, School of Public Health, Addis Ababa University, Addis Ababa, Ethiopia

## Abstract

Street children worldwide do not have the information, skills, health services, and support they need to go through sexual development during adolescence. This study is undertaken to systematically investigate the fit between street children's sexual and reproductive health needs and the existing services. A cross-sectional study was conducted among 422 street children and four service providers. About 72.5% of the respondents were sexually active during data collection and 84.3% of males and 85.7% of females tended to have multiple sexual partners. More than two-thirds (67.3%) of the participants had used at least one type of substance. History of substance use (OR = 2.5; 95% CI = 1.42–4.56) and being on the street for the first one to three years (OR = 5.9; 95% CI = 1.41–7.22) increased the likelihood of having sexual activity. More than half (64.9%) of the street children did not attend any kind of sexual or reproductive health education programs. Lack of information on available services (26.5%) was the biggest barrier for utilization of local sexual and reproductive health services. From the individual interview with coordinator, the financial and networking problems were hindering the service delivery for street children. In conclusion, street children who are special high risk group have not been targeted and hence continue to remain vulnerable and lacking in sexual and reproductive health services and sexual health services are poorly advertised and delivered to them.

## 1. Introduction

The problem of street children is becoming a worldwide phenomenon since these children exist in every part of the world. The vast majorities of these children work and live in large urban areas of developing countries [1]. Like many underdeveloped nations challenges facing the Ethiopian children are diverse and immense. Thousands of children live under difficult circumstances and are exposed to various forms of abuse and exploitation [2]. Street children in this study include those children aged between 10 and 18 years. The family may have neglected them or may have no family members left alive. Such children have to struggle for survival and might move from friend to friend or live in shelters such as abandoned buildings, plastic shelters, and public phone rooms [3].

Though street children are hard to count, estimates of the number of street children range from about 20 million to over 100 million worldwide. What is certainly known is that their numbers are increasing for various reasons including the global population growth, poverty, rapid urbanization, and AIDS pandemic [4].

In Ethiopia, due to push factors (poverty, family dysfunction abuse, and school problems) and pull factors (independence, freedom, and drug/alcohol abuse) children are drifted to street life to support themselves or their families in major cities [5]. Over 4 million children are estimated to live under especially difficult circumstances. It is estimated that 600,000 children are taking part in street life and as many as 500,000 children find themselves at an extremely high risk of becoming involved in street life in Ethiopia [6].

The streets of Addis Ababa, the capital city of Ethiopia, are said to be home to a population of between 60,000 and 100,000 street children with the lower estimates originating from the Ministry of Labor and Social Affairs and the higher ones from aid agencies [7]. Street children live and work in conditions that are not conducive for healthy development. They are exposed to the street subculture such as smoking, drug, alcohol and substance abuse, gambling, engaging in sexual activities, or selling sex for survival [1]. The circumstances in which they live and work increase their vulnerability also to sexual exploitation and abuse and put them at a higher risk of unintended pregnancies, sexually transmitted infections and HIV/AIDS. The problem is further compounded by the lack of access to sexual and reproductive health information and services [8]. A few studies that exist on the sexual behavior of street children in Addis Ababa showed that these children are more familiar to high-risk behavior and are sexually active at an early age [6]. Despite these alarming realities, street children rarely have a voice in the sexual and reproductive health discourse. Governmental and nongovernmental organizations intervention programs do not based on the realities of street children. This is because these organizations work through the existing societal structures such as hospitals, schools, local communities and facilities from which street children are disconnected [4].

Though study reports and other literatures on street children sexual health problems are very limited and even when available are not comprehensive, some studies tried to reveal the magnitude of sexual and reproductive health problems of street children andtried to recommend appropriate intervention strategies for governmental and nongovernmental organizations [6]. To date, little is known about sexual and reproductive health status of street children and whether they have access to sexual and reproductive health services and information and, if so, to what extent. Hence undertaking a study in this area is believed to provide information on their sexual and reproductive health status and the types of sexual and reproductive health services offered to street children and relevant information was generated that could help organizations to design appropriate sexual and reproductive health programs and improve future services for this disadvantaged segment of the population.

## 2. Literature Review

### 2.1. Background

A personal sense of sexual wellbeing as well as the absence of disease, infections, or illness associated with sexual behavior is termed as *sexual health*. People with adequate sexual and reproductive health have a satisfying and safe sexual life, can have children, and can make a choice as to whether they would like to have children and, if so, when and how to have them [9]. Unfortunately youth in general are at greater risk for abnormal sexual and reproductive health compared to other age groups due to risky sexual behaviors. Homeless children and youth are likely to be at even higher risk for several different reasons [10]. A risky sexual behavior is one that increases the likelihood of adverse sexual and reproductive health consequences. These health consequences may include unwanted pregnancy, unsafe abortion, HIV/AIDS and STIs [9].

### 2.2. Risky Sexual Activities

#### 2.2.1. Sexual Activity under the Influence of Substance/Alcohol

Substance use may influence sexual behavior in ways that increase the risk of acquisition of HIV and other STDs. The street child's decisions on sexual behaviors such as whether to use a condom during sexual activity, to negotiate for sex, or to use force (rape) depend on the level of intoxication. In general alcohol and other substance use often accompany the early sexual experiences, especially among boys [9, 11].

#### 2.2.2. Commercial Sex/Survival Sex/Prostitution

It has been estimated that 25% of Ethiopia's street children are girls and there is indisputable evidence that street girls in Ethiopia are often obliged to take on commercial sex work for survival. Unfortunately recent information on the number of street girls engaged in it is lacking [1]. One of the studies done in Addis Ababa showed that a significant number, 69.6%, of the study subjects (who were commercial sex workers) were between the ages of 13 and 15 years [12].

#### 2.2.3. Unprotected Sexual Intercourse

Unprotected sex is common among street children. This could result in a variety of sexual and reproductive health problems. Street children spend a lot of time in settings where casual sexual encounters occur (bar or “crack houses”) [11]. Their risk of acquiring blood borne diseases and STDs such as HIV, syphilis, and hepatitis is increased by the fact that they often have sex with persons at high risk for these diseases like people with multiple sexual partners or those sharing injection equipment for substances. Research results highlight the critical need for sexual and reproductive health programs for street children. For example, a study in Awassa showed that, Among the 280 respondents who practiced sex, 216 (77.1%) did not use any of the modern methods of contraception [13].

#### 2.2.4. Same Sex Sexual Activity

Street children sometimes have sex with other street children of the same sex. This is much more common among boys. In addition, street boys are often sexually exploited by older men. Engaging in unprotected sexual intercourse can lead to acquisition of STDs including HIV [9]. Based on the study done by Getnet Tadele on sexual abuse against male street children in Merkato area, Addis Ababa, peers/friends were found on the top list of abusers as reported by 47% of the children, followed by unknown persons/strangers 17%, relatives 11%, and 10% students and rich businessmen. Foreigners/Ethiopian diaspora, bar owners, and police were reported by 6%, 5%, and 4% of the respondents, respectively. This finding shows that street children themselves are involved in the practice [14].

### 2.3. Consequence of Risky Sexual Behaviour and Unprotected Sex

#### 2.3.1. Pregnancy and Its Consequences

Street girls may become pregnant because of unprotected sex and the baby born to such mothers may have a low birth weight and may be prone to infections and illness. Coping with the needs of the child may be difficult for a street girl [15]. Although having a child before the age of 15 is reported to be common in many families, unwanted pregnancy in early age is a complicated process and it accounts for the majority of maternal mortality and morbidity [12]. One study conducted in Dessie town showed that, out of sexually active female street youth, 25.0% had a history of unintended pregnancy at least once prior to the study, out of which 55.5% of them reported history of induced abortion at least [16]. Another study in Addis Ababa revealed that nearly a quarter (23%) of the girls had encountered unwanted pregnancy meaning that, in one way or another, they were exposed to unprotected sex (either consensual or forced). It, therefore, suggests that unprotected sex among commercial sexual workers is not rare phenomenon. Girls who faced unwanted pregnancy in the same study were also asked about the measures they took to deal with the problem and over half (57.2%) reported that they terminated it using unsafe/traditional means of abortion, 17.8% terminated it in a clinic/and other medical institutions while a quarter of them (25%) opted to deliver the baby/instead of abortion [17].

#### 2.3.2. Sexually Transmitted Infections and HIV

Sexually transmitted infections including HIV are consequences of unprotected sexual intercourse with an infected individual. The risk of STIs increases if a person has more than one sexual partner [9]. A study conducted by Taffa showed an overall HIV-1 prevalence of 5.3% among the 358 out-of-school youth in Addis Ababa. There was a 60% excess prevalence rate among out-of-school females compared to that among the males. The study indicated significant prevalence of HIV infection, particularly among female and out-of-school youth [17]. Available data showed that HIV seroprevalence rates for street children are 10–25 times higher than that among other groups of adolescents [18].

### 2.4. Street Children's Sexual and Reproductive Health Needs

#### 2.4.1. Accurate Information

Information lies the basis for interventions that follow, such as the building of skills and counseling. Street children should be provided with information on growth and development, sexual and reproductive health, substance use, prevention of disease, promotion of good health and other issues such as rights and laws [15]. When working with street children, it may not be possible to find an appropriate place or time to provide information to them. It is important to take advantage of as many situations as possible whenever in contact with street children.

#### 2.4.2. Life Skill Training

Life skills are positive behaviors that enable individuals to adapt to and deal effectively with the demands and challenges of life [15]. It can also help street children in taking the opportunity to get off the street. Helping street children think about strategies for getting off the streets will need to include creative ways of getting them to think beyond their current situation [19].

#### 2.4.3. Safe and Supportive Environment

The term environment is used to refer to what a child encounters outside of himself or herself in daily life. It refers to the political, legislative, legal, economic, social, and cultural context of the child's life, including opportunities to get an education and gain livelihood skills as well as the opportunity to experience positive relationships with other people. This broader environment influences behavioral choices. The aim of creating a safe and supportive environment is to promote positive behavior among street children [15].

Unless immediate preventive and protective measures are taken to check the spread of the problem, it will greatly endanger the rights of survival and development of children protected in the convention of the right of children (CRC) [20]. It is only by recognizing the barriers and limitations imposed upon them by mainstream society as a whole and health services in particular, that their needs can be met [21]. But homeless people feel excluded from mainstream sexual health services. Numerous real and perceived barriers exist, which make it difficult for them to engage fully with these services. Many street children may not also consider sexual health to be a priority. This in itself can make providing a sexual and reproductive health service for this group a difficult task [1]. In order to tackle these problems, government and NGOs should provide both internal and external resources. Internal resources like intelligence, capacity to work and external resources in the environment like schools, health services, community organizations, and people who care can induce positive impact on street children's life. Even though street children usually have many internal resources, they usually lack external ones [15]. Life skills (the ability to be assertive about choices of sexual activities and to negotiate the use of contraceptive method) can help street children resist sex or have safer sex. Similarly, life skills such as problem solving and critical thinking help in healthy decision making. Practical skills like knowing how to use a condom are essential for the practice of safer sex. Livelihood skills would decrease dependence on survival sex [22]. Street children are also traditionally reluctant to access health services due to transportation problems along with a perceived lack of respect from providers and fear of being judged by health care workers, creating physical and psychological barriers to accessing health care. Surveys of homeless youth have found that health advice is most often required from other homeless persons, followed by self-treatment, and finally accessing clinics when self-treatment no longer work [23].

It is also possible that support-giving organizations are not publicizing their services sufficiently. This is due to the fact that the capacity of these organizations is extremely low in comparison to the number of street children in need of sexual and reproductive health support [24].

In the assessment survey done for supports provided to street children, respondents were requested to indicate whether or not they have knowledge of support-giving organizations and only 36.7% said that they did with the proportions being 58.5% for female and 34.1% for male within gender groups, respectively. This shows that street children, especially male street children, even in Addis Ababa have low access to the public media including the radio, TV, and newspapers. If these children had some access, they would have known at least one or two organizations working in support of street children [6]. Respondents who reported that they were getting services from support providing organizations were further asked to specify the type of support they were receiving and the highest proportion (44.8%) reported that they were getting support for food. This shows that sexual and reproductive health issues for street children are neglected [6].

Given the numerous reasons associated with poor sexual and reproductive health among street children like histories of abuse and sexual risk-taking behavior, a holistic approach to intervention is necessary to improve their wellbeing.

But still the problem of street children remains an ignored tragedy. Street children are not targeted in fighting against HIV/AIDS [5]. For example, in one study, When the key informants were asked about the attention given to the problem by governmental and nongovernmental organizations, they all unanimously replied that enough has not been done [14].

This study therefore tries to systematically investigate street children's Sexual and reproductive health status and services, their utilization patterns, and street children-friendliness from the point of view of street children and service providers. The study also endeavor, to pinpoint the existing problems and gaps in providing sexual and reproductive health prevention and treatment services for street children. Such findings can hopefully impact the manner by which service provider can address sexual and reproductive health needs effectively.

## 3. Objectives

### 3.1. General Objective

To assess the sexual and reproductive health status of street children in Addis Ababa.

### 3.2. Specific Objectives

- To assess sexual and reproductive health needs of street children in Addis Ababa.
- To assess the nature and scope of sexual and reproductive health services for street children in Addis Ababa.
- To determine the proportion of street children who are aware and have used specific sexual and reproductive health services.
- To examine factors associated with sexual activity.

## 4. Methods

### 4.1. Study Area

The study was conducted in Addis Ababa the capital of Ethiopia which has an area of 530.14 square kilometers divided into 10 subcities (Kifle Ketema) with a total of 100 kebeles. The study was carried out from December 29, 2010, to January 13, 2011. Based on 2007 Ethiopian census, Addis Ababa has a total population of 2,738,248, consisting of 1,304,518 men and 1,433,730 women [25]. Addis Ababa is the largest urban area in Ethiopia and attracts many children and youth who are searching for employment. The city has a high population of street children who are engaged in the informal sectors. The research was conducted in the five sub cities of Addis Ababa: Arada, Addis Ketema, Kirkos, Lideta and Bole. These sub cities were selected purposively for the research based on high concentration of street children.

### 4.2. Study Design

Since triangulation of research methods can overcome personal biases and limitations that stem from the use of a single method, cross-sectional quantitative and qualitative mixing methods were used in the current study. Individual interviews using a structured questionnaire were conducted to gather relevant information on sociodemographic characteristics, substance abuse, sexual behavior, sources of information on HIV/AIDS and unwanted pregnancy (Table 9), and so forth. Qualitative methods were then conducted to verify data collected in quantitative method and to gain an in-depth understanding of the sexual behaviour and the service provided to them.

### 4.3. Study Population

The source populations for this study were street children in Addis Ababa and street children, who were living or working independently on the street, aged 10–18 years, resided in Addis Ababa for at least 6 months, and can speak and heard Amharic language, were the study population of this research.

### 4.4. Sample Size

Considering the absence of previous data in Ethiopia in this specific study group which comprises both sexes up to the knowledge of the investigator and to obtain a large sample size the following assumptions are undertaken. The proportion of street children having at least one type of sexual and reproductive health serviceis estimated to be 50%, with a precision level 5% and 95% confidence interval. 10% was added to compensate for nonresponse. Based on this assumption, the actual sample size for the study was computed using the formula for single population proportion as follows:

(1) $$\begin{array}{c} n=\frac{{\left(Z\alpha /2\right)}^{2}p\left(1-p\right)}{d^{2}}+10\%\;\;\text{non}\;\;\text{response}, \end{array}$$

where *n* is sample size, *p* is expected proportion (0.5), and *d* is margin of error/level of precision (0.05). Therefore,

(2) $$\begin{array}{c} n=\frac{{\left(1.96\right)}^{2}0.5\left(1-0.5\right)}{{\left(0.05\right)}^{2}}=\frac{\left(3.8416\times 0.25\right)}{0.0025},\\ n=384. \end{array}$$

Thus the study included 384 study subjects plus 10% nonresponse. Then the data was collected from 422 street children.

### 4.5. Sampling Procedures

In Addis Ababa, street children are known to congregate in various city locations throughout the day. These locations, along with time at which high number of children congregated, our sampling frame. Based on the calculated sample size and determined the number of locations needed. In this case the sample size was determined to be 422 and during the formative assessment, the minimum number of children found in each location was 10 therefore 42 locations were considered. In order to create the frame we observed the sites and counted how many street children were there for a specific time of day (1 to 2 hours). An earlier work done by Forum on Street Children-Ethiopia (FSCE) was used as base for sampling plan [6]. This earlier work was adapted to suit this work on demanded information. Finally we selected 42 of the venues randomly from the universe of venues.

### 4.6. Sample Selection

A sampling frame of locations which were defined by both location and time was constructed. Locations and individuals in that location were selected using equal probability sampling (lottery method). The interviewers were instructed to try a take-all approach. They were only going to each site once and got everyone they could in the time period 2 hours no more or less. In this case any one that was present had an equal chance of being interviewed.

### 4.7. Data Collection Procedures

The data for the quantitative section of the study were collected by 10 trained data collectors (8 males and 2 females) for 14 days who were master students in public health at Addis Ababa University with some experience in data collection in previous studies. To maximize openness of the children male interviewers were assigned for male respondents and female interviewers were assigned for female respondents. A structured questionnaire extracted from standardized questions such as BSS which addressed all the variables, was prepared and pretested. The pretest was conducted among 25 street children and these were excluded from the study. The data collection was conducted within two weeks from December 29, 2010 up to January 13, 2011.

The second set of instruments constitutes unstructured questions designed to serve as a guide for focus group discussion and interview with service providers. A total of four (two among males of 10–14 years of age and 15–18 years of age and another two among females of the same age group) focus group discussions were conducted in the two sub cities (Arada and Addis Ketema). FGDs were conducted separately for boys and girls. Each FGD was consisted of 8 participants. Participants were selected in such a way that children do not know each other to encourage them to expresse the realities.

### 4.8. Data Analysis Procedures

The quantitative data was entered into EPI Info version 3.5.1, then exported to SPSS version 16 statistical program. Descriptive statistics of percentages mean and frequency distribution using tables and figures were carried. In addition Bivariate analysis was used to determine the association between different factors and sexual activities. Those variables which have significant association with sexual activities were entered to multivariate analysis. Finally binary logistic regression and odds ratio with 95% confidence intervals was used to identify the independent predictors of sexual activity. FGD notes were typed and the audio tapes were transcribed. Responses were analyzed by arranging them in the general categories identified in the discussion guide. The various opinions were assessed and summarized so that the degree of consensus or differences were expressed by the groups and synthesized by the themes or patterns that emerged. All the recorded interviews with service providers were transcribed and analyzed manually.

### 4.9. Data Quality Management

The quality of data was maintained through careful design; translation, and retranslation, and pretest of the questionnaire, proper training of the interviewers and close supervision of the data collecting procedures; and proper categorization and coding of the data.

### 4.10. Study Variables

*Dependent Variable*. Sexual practice.

*Independent Variable*. Sociodemographic and economic variables (sex, age, and previous residency), connectedness to NGOs, and life skill training and personal factors like smoking status, khat, and alcohol use.

### 4.11. Operational Definition

*Street Children*. They are children less than 18 years old, comprising *on* and *off* street children.

They are children in difficult circumstances, who struggle to survive in the city.

*Children on the Street*. Those children who primarily engaged in economic activities of street. They are children of either sex falling with the age group of less than 18 years working or begging on the street but living with their parents or visiting their parents regularly.

*Children off the Street*. Children of either sex who are within the age group of less than 18 years and who are both economically and socially engaged in street life. These children live and work on street without any kind of control or assistance from parents or relatives.

*Risky Sexual Practice*. Children who had sex earlier than 18 years of age, or have sex with nonregular sexual partner, or exchange sex for money, or have more than one sexual partner or use condoms inconsistently.

*Rape*. It is defined as any nonconsensual of penile penetration of the vagina or anal by physical violence or by threat of harm, or when the victim is incapable of giving consent due to drug or intoxication of alcohol.

*Drug/Substance*. Any substance that when taken into the living organism may modify one or more of its function. In this study the concept of drug covers substances of alcoholic drinks, tobacco, khat, hashish, and benzene.

*Sexual and Reproductive Health Needs*. They include access to health care, services, sexuality education, and access to birth control method.

*Organizational Response*. Any organized primary prevention, care or support activity designed by many actors to make sexual and reproductive health information and services available to street children.

### 4.12. Ethical Consideration

In this study, parents/guardians were not available. Therefore children were asked personally for their consent to participate. All participants were given full information regarding the purpose of the research, what is expected from them and how long the interview is expected to last. In addition the research did not expose children to any physical and emotional stress. Furthermore the study protocol was approved by the Addis Ababa University, College of Health Science, Shool of Public Health Research and Ethics Committee (REC). The objective of the study was also discussed with organizations that are working with children and Addis Ababa police crime protection sector (child protection unit, CPU).

## 5. Results

In total, 422 street children, 314 (74.4%) males and 108 (25.6%) females were interviewed resulting in an overall male to female ratio of 3 : 1. As indicated in Table 1, from the total of interviewed street children, 73.2% were “off the street” type while the rest were “on the street” type. The age range of those children included in this study was between 10 and 18 years. Nearly two-thirds (65.2%) of the sample street children respondents were between 16 and 18 years of age and 31.0% are between 13 and 15 years of age while only 3.8% of the children are between 10 and 12 years of age. The mean age was 15.9 (SD ± 1.7) years. The mean age for males and females was 15.9 years and 16 years, respectively. Street children were also asked how long they had been on the streets and roughly half (51.2%) indicated that it had been from 1–3 years while quarter (24.9%) of them said less than one year and 15.9% for 3–5 years. Fewer than ten percent (8.1%) had been on the streets for more than five years. Furthermore, majority street boys (81.5%) than street girls (24.1%) were sleeping on the street during the night. Up to 77.9% of participants had dropped out-of-school at the primary level. More than two-fifths (44.3%) were dropped out from 5 to 8 grades and 33.6% from 1 to 4 grades while only 1.9% were from 9 to 12 grades and 20.1% were either never attended or read and write only.

Regarding income generating activities, nearly all participants (94.1%) were involved in an income-yielding activity (Table 2). The sources of income for males and females were different. Males were mostly carrying items (54.1%), washing and watching cars (10.8), doing any occasional jobs (10.5%), and shoeshining (7.6%) and 7.0% were involved in other activities like jeblo (cloth selling), hair dressing, stealing and so forth, while females (43.5%) were involved in commercial sex and 16.7% were doing any occasional jobs while 15.7% were working as message conveyer. Concerning the average income they earned per day, most of the participants (36.8%) earned 11–20 birr and 33.0% interviewee indicated 5–10 birr per day while only 10.8% stated that they earned more than 50 birr per day (majority were female commercial sex workers.

### 5.1. Reasons for Joining the Street

According to the responses of the children involved in the study, job searching account for about 28.9% (26.8% males versus 35.2% females) and peer influence are the second most common reason for their initiation of street life which accounts for 21.8% (19.4% males versus 28.7% females); family disharmony 19.2% (19.4% males versus 18.5% females), orphaned 18.5% (20.1% males versus 13.9% females), poor family 4.3% (5.4% males versus 0.9% females), and alcoholic parents 3.3% (4.8% males versus 1.9% females) are mentioned by the respondent as reasons for joining the street life. The above reasons push children mainly boys to engage in street activities.

**Figure 1:**
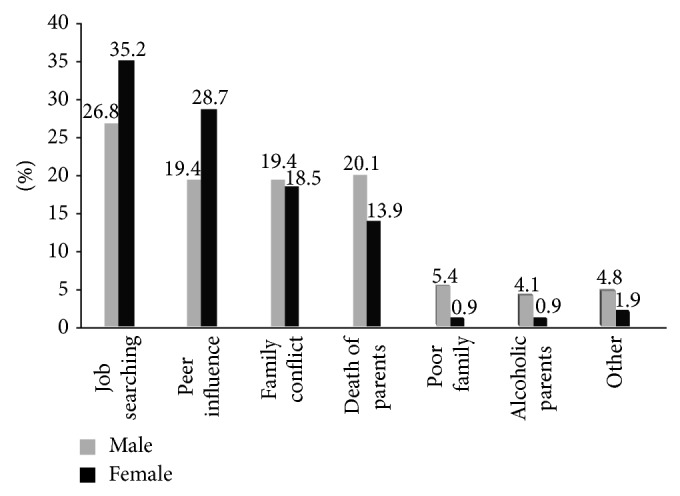
Reasons of street children to join street life in Addis Ababa, January, 2011.

**Figure 2:**
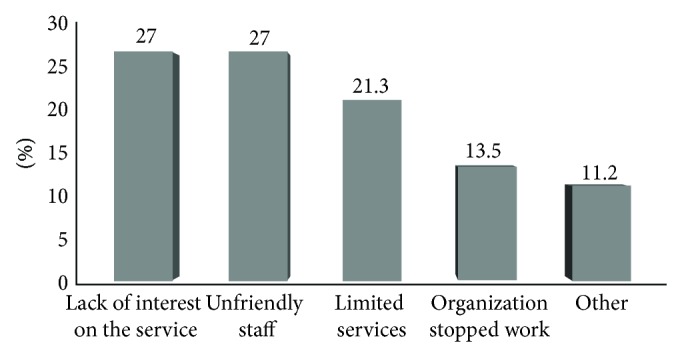
The distribution of street children by their reasons for rejoinig the street life in Addis Ababa, January, 2011.

In our interviews almost one-third of street children (33.4%) claimed to have been supported by one organization at least once, but they had left and come back to the streets (Figure 1). The remaining 46.7% never been helped and 19.9% were using the service (mainly food) during data collection. Children were further asked why they came back to the street. Respondents had diverse reasons for rejoining the street (Figure 2). Among the reasons, the services were not based on our interest and unfriendly staffs were equally mentioned by 27.0% of the respondents each. While 21.3% of them stated limited services as a reason for rejoining the street life and 13.5% mentioned the organization stopped its work now, the remaining 11.3% of street children mentioned other reasons like finishing their training term, conflict with other children, long distance, and so forth.

The street children in Addis Ababa are just as diverse as any other group in society. Almost three-fourths of interviewed children (70.9%) had arrived to Addis Ababa from other towns and regions of the country. 36.1% of girls and 26.8% of boys were born in Addis Ababa and 63.9% of girls and 73.2% of boys came from other regions or towns of the country.

### 5.2. Substance Use

Data received through the interviews indicated that 284 (67.3%) of the selected sample of street children consumed various substances or drugs on a habitual basis, whereas 138 (32.7%) did not refer to use at the time when interviews were conducted (Table 4). Gender differences were not found to be highly significant for substance use. Overall, the findings indicated that almost two-thirds of street children in Addis Ababa used one or more substances. Substance abusing was further investigated by requesting respondents to indicate the type of substances they were using. According to the results summarized in Table 3 multiple responses were forwarded. It was found out that, among those who were used, nearly all (95.1%) of them chew chat, while three-fourths (75.7%) smoke cigarette, one-fifth smok shisha and (6.0%) sniff benzene. Comparison by gender revealed that within gender group more boys (81.3%) than girls (40.0%) smoked cigarettes while more girls (98.7%) than boys (93.8%) were chewing chat.

**Figure 3:**
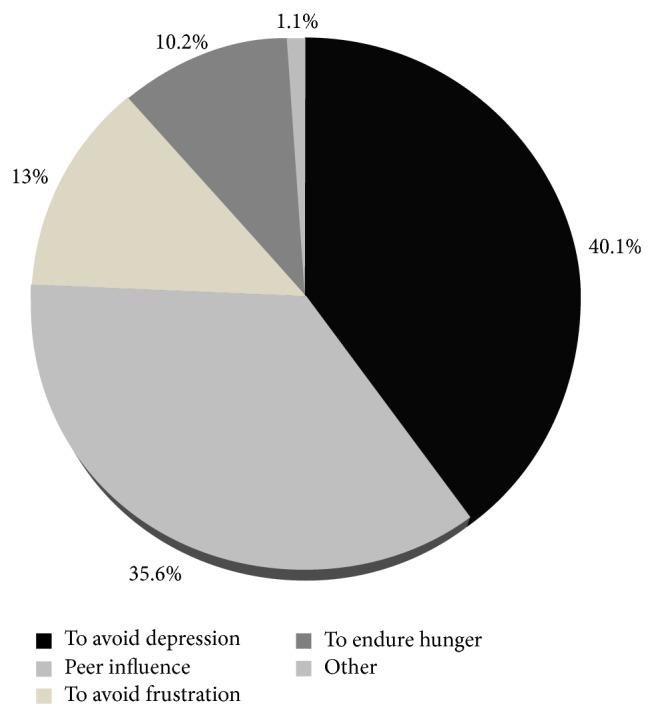
Percentage distribution of reasons for substance use among street children in Addis Ababa, January, 2011.

Substance abuse was further examined by requesting respondents to identify the reasons that initiated them to use the various substances. The findings are summarized in Figure 3; majority (40.1%) of street children started substance use to avoid depression, while 35.6% of them initiated it by peers, 13% stated to avoid frustration during sex or stealing, and other 10% of them stated to endure hunger.

### 5.3. Alcohol Intake

Alcohol consumption of street children was investigated by requesting respondents to indicate their exposure to alcohol drinking (Table 5). Accordingly the multiple responses of the respondents indicate that among interviewed children, almost two-thirds (64%) of the respondents were drink alcohol. The frequency of their consumption were tried to investigated. Of those 50.0% drunk sometimes (once per week) and 9.3% drunk most of the time (three times per week) while 4.7% drunk daily. Interviewed children were further asked for their sexuality after alcohol intake. Their responses as given in Table 4 indicated that 177 (65.6%) of them have sex after alcohol intake most of the time. Further analysis by gender revealed that females had sex after alcohol more frequently than male street children (83.5% versus 59.4%). Significant number of children (66.7%) used condom during sex after alcohol intake. But 15.3% of the street children did not use condom while 18.0% of them responded as they do not remember due to heavy intoxication.

**Figure 4:**
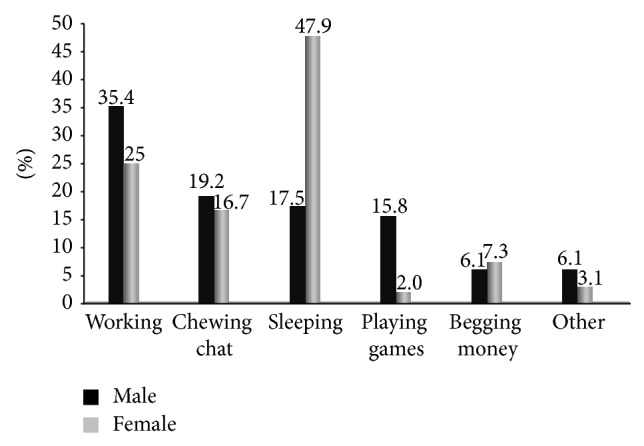
Common day time activities by street children in Addis Ababa, January, 2011.

Children were asked how they spend most of their day times. High proportion of them (32.8%) responding working (35.5% males versus 25.0% females), sleeping 24.9% (17.5% versus 47.9%), and chat chewing 18.6% (19.2% versus 16.7%) (Figure 4). The reason for significant differences in this regard between males and females specially for sleeping could be the presence of high number of female street children working during the night as commercial sex worker.

### 5.4. Risky Sexual Behavior

The majority of street children respondents 302 (71.6%) said that they had ever practiced sexual activity (65.0% of boys and 90.7% of girls). The overall mean age at first sexual intercourse is 15 years with male mean age at first sexual intercourse 15.4 (SD ± 1.2) years and female 14.3 (SD ± 0.87) years. Among sexually active street children 42.7% mentioned personal desire as a reason for having sexual intercourse. Other reasons for sexual intercourse are fall in love 34.4%, exchange for money 8.3%, peer pressure 7.0%, marriage 3.3%, influence of chat/alcohol 2.3%, and rape 2.0%.

Sexually active street children were also asked about their sexual experience within the last three months. A total of 197 (65.2%) respondents answered as they had sexual intercourse at least once. They further asked about whether they faced unwelcome sex within the last 12 months and about 55 (18.2%) responded that they have faced unwelcome sex. Out of these victims 23 (41.8%) were males and 32 (58.2%) were females.

Children were also asked to mention risky sexual activities which they expect expose them to sex related problems. Majority of respondents 129 (30.6%), answered that they did not remember and 101 (23.9%) mentioned sex without condom. Injury with sharp materials was indicated by 67 (15.9%) children. Other activities like more than one sexual partner 58 (13.7%), sex with commercial sex worker (male only) 33 (7.8%), inconsistent condom use 17 (5.0%) were mentioned as risky activities that were performed previously.

To identify the current most common sexual and reproductive health problems in the street life, children were asked questions related to sexual and reproductive health problems in the last 12 months before the survey. More than half of street children 233 (55.2%) respondents (199 (63.4%) males and 34 (31.5%) females) answered that they faced no problem, while 198 (44.8%) have encountered sexuality related problems. Among commonly mentioned sexual health problems, unprotected sex under the influence of chat/alcohol 60 (14.2%), unwanted pregnancy 40 (9.5%), rape attempt 35 (8.3%), STIs 24 (5.7%), rape 23 (5.5%), and abortion 7 (1.7%) were indicated as the main sexual related health problems.

Concerned with life time number of sexual partners 67.6% have more than one sexual partner and 17.2% responded as they cannot remember the number of individual that having sex with them. These are particularly male children who have sexual relation with commercial sex workers and female children mainly involved in commercial sex, while only 15.2% have a single sexual partner.

### 5.5. Pregnancy among Female Street Children

Among the 108 street girls who participated in this study, more than half (70.4%) reported that they had ever been pregnant (31.9% once and 68.1% more than one time) and almost three-fifths (59.4%) of these pregnancies resulted in abortion. Nearly all (95.7%) of female respondents said that pregnancies were unwanted. Further the reasons for pregnancies were asked and reluctance to use contraceptive (42.4%), unavailability of contraceptive (16.6%), inappropriate use of contraceptive (15.2%), rape (10.6%), and others like slippage of condom, failure of contraceptives (15.2%) were mentioned as the main reasons for the occurrence of unwanted pregnancies.

From the analysis it was found that some sociodemographic and other variables were significantly associated with practicing sexual intercourse of participants. A history of drug use (OR = 2.5; 95% CI = 1.42–4.56) and being on the street for the first 1–3 years (OR = 5.9; 95% CI = 1.41–7.22) increased the likelihood of having sexual activity. Street boys (as compared to girls) are significantly less likely to report having sex (OR = 0.09; 95% CI = 0.03–0.23) preceding the survey. Alcohol use is significantly associated with engaging in sexual activities (OR = 5.2; 95% CI = 3.73–8.32). Sexual behaviour of street children is also associated with their former residences. Street children who reported that they were coming from outside of Addis Ababa were more likely to experience sexual activities (OR = 1.7; 95% CI = 1.10–2.70) and children who had no connections with NGOs (through infrequent or even frequent visits to the NOGs) show most likely to be engaged in sexual behavior (OR = 2.1; 95% CI = 1.25–3.64) (Table 8).

Regarding the sexual and reproductive health information received Table 6 shows that a modest proportion of street children 191 (45.3%) of the total interviewed responded that they have information about the issue (sexuality, STIs, and HIV/AIDS), while regarding gender difference, Boys received less information 134 (42.7%) regarding prevention with pregnancy, STDS, and HIV/AIDS compared to girls 57 (52.8%).

Participants were further asked about their source of information about pregnancy, STIs, and HIV/AID (Table 10). The majority (28.8%) stated mass media, 19.4% stated friends (other street children), 16.8% stated health workers, 11.0% stated schools, (before dropping out-of-school), 8.4% stated outreach workers (street educators), 6.8% and others like training and posters, and pamphlets.

The study questionnaire also includes questions to identify the barriers of health information for street children. As a result, those children responding have no information about pregnancy, STIs, and HIV/AIDS were asked to mention the reasons. The significant numbers of street children were answered inaccessibility 94 (40.7%) as the main reason for lacking information. Other reasons, less priory 79 (34.2%) and it does not concern me 58 (25.1%) were mentioned.

On the other hand, older street children are more likely to receive such information as compared to their younger counter parts. only 10.5% among 10–12 age group and 39.1% within 13–15 age group have informed, while half of the 16–18 age group have reported that they have received such information.

The study participants were also asked about the reliable and easily accessible health information sources about unwanted pregnancy, STIs and HIV/AIDS for street children. Majority of participants 194 (46.0%) were mentioned their friends as the main reliable and easily accessible information source followed by street educators 98 (23.2%).

**Figure 5:**
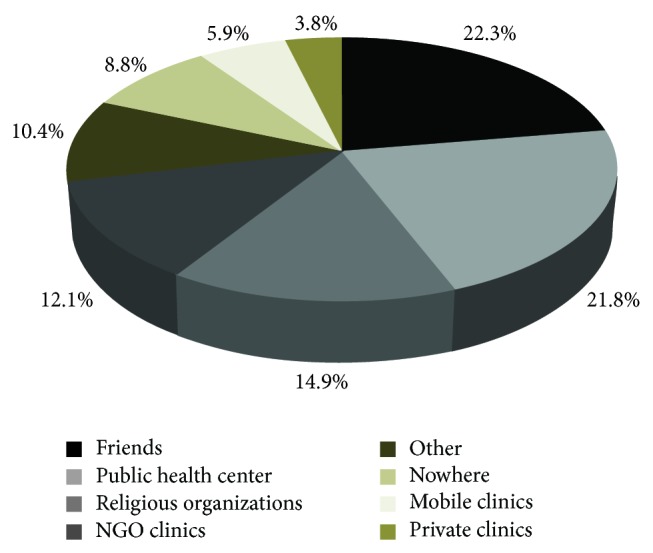
Distribution of street children by preferable place for sexual and reproductive health services and advices in Addis Ababa, January, 2011.

In relation to places where they go for help when sexual and substance abuse related health problems faced, (22.3%) of participants stated friends, 15% stated public health centre, 14.9% stated religious organizations, 12.1% stated NGO clinics, and 8.8% responded that they do nothing, 5.9% stated mobile clinics and only 3.8% mentioned private clinics (Figure 5).

Study participants were further asked for the reasons why they prefer these places. As result they list their reasons by mentioning the majority 31.0% reported they easly understand my problem as the main reason followed by 22.8% stated no other alternatives, 14.7% free service, 12.2% short waiting time, 10.2% good confidentiality, 6.6% affordable cost and 2.5% others like short distance, friendly staff and so forth (Table 13).

The interviewers also ask the street children about the current most common sexual and reproductive health problem for street children. Rape was mentioned by the majority of street children 105 (24.9%) as the most common sexual health problem for street life.

Life skills education programs that include sexual and reproductive health information have proven to be effective in delaying the onset of sexual intercourse and, among sexually experienced children, in increasing the use of condoms and decreasing the number of sexual partners. This reality was observed in this study. Significant difference was observed among participants who took life skill training and who did not on the knowledge of SRH service providers (43.9% versus 20.5%) (Table 11).

To identify the level of awareness of the street children about the health centers that exclusively give sexual and reproductive health services for street children the questionnaire included the following question: “do you know any health facility that provide sexual and reproductive health services for street children?” Only 24.2% of the participated street children have heard about such health facilities. The remaining 75.8% of interviewed children responded that they have never heard about them out of which (48.1%) stated that there are no such facilities and (27.7%) were not sure (might or might not) of the presence of such health facilities in Addis Ababa (Table 12).

The analysis of the responses by sex of the respondents led to even more alarming results. Female children more often than male children have stated that they have heard about the health centers (36.1% among females versus 20.1% among males).

Furthermore there was a question for those children who have heard/know health facilities that provide sexual health for them about the type of health facility they know. 38% of them mentioned family guidance and 30.4% stated NGO clinics. Of the remaining mentioned 12.0% stated public hospital, 7.8% public clinics, 5.9% privet clinic and 4.9% others like traditional.

The next question was about their experience of using theses health facilities and almost half (47.1%) of the street children who have heard about these health facilities have actually visited them for service. During the data analysis a slight correlation between the type of answers and the gender of the respondents was identified 63.2% among females and 37.5% among males have visited the health center.

**Figure 6:**
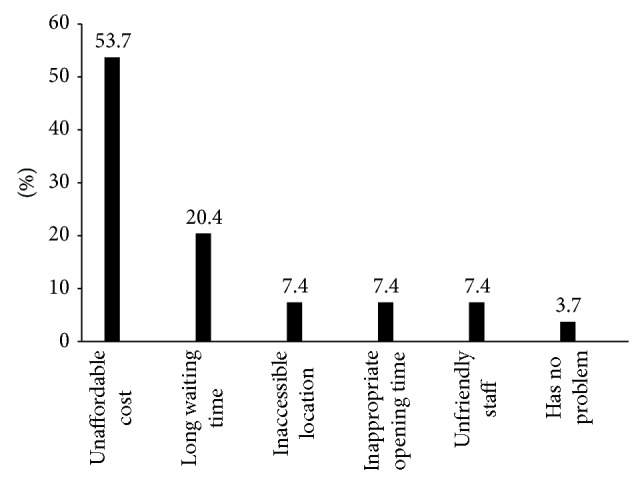
The distribution of street children due to the reason that inhibite visiting the health facilities in Addis Ababa, January, 2011.

The remaining 52.9% were asked to identify any barriers they faced when accessing services from the above health facilities. More than half of the respondents (53.7%) mentioned unaffordable cost as the main reason for not visiting the health facilities. while 20.4% stated long waiting time and inaccessible location, unfriendly staff, for inappropriate opening time each accounts 7.4% and I have no problem accounts for about 3.7% (Figure 6).

Street children's opinion about the sexual and reproductive health services given to them in Addis Ababa was asked and the responses from the participant are given in the following table. Based on the respondents answer, for each sexual and reproductive health service characteristics, majority of them were answer as not fulfil friendly service character.

**Figure 7:**
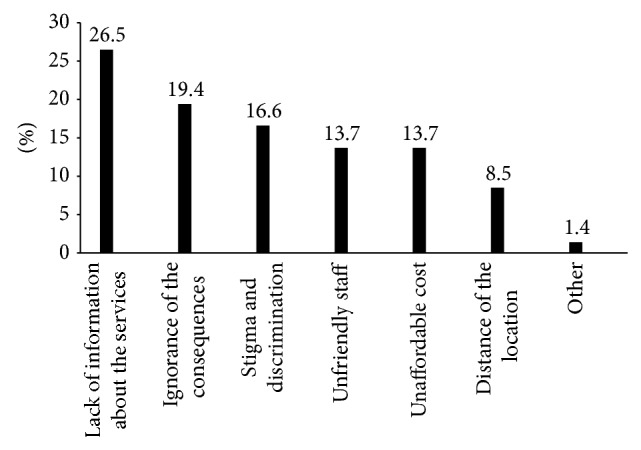
Barriers for street children utilization of local sexual and reproductive health services in Addis Ababa, January, 2011.

Participants were also asked what barriers they faced when accessing local sexual health services (Figure 7). The majority, 26.5% of participants stated lack of information on available services as the biggest barrier, 19.4% stated ignorance of the consequence of risky sexual activities, 16.6% stated fear of stigma and discrimination, 13.7% stated unaffordable cost and 13.7% stated lack of unfriendly staff. The participants also were able to state “other” barriers which account for about 1.4% of respondents.

**Figure 8:**
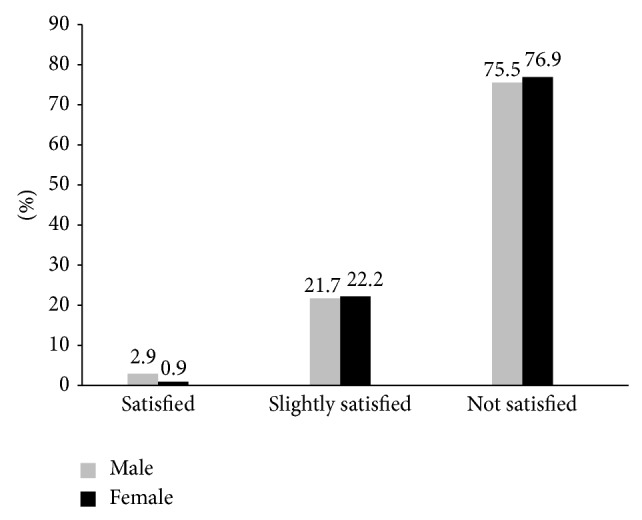
The distribution of street children by their satisfaction status with SRH services in Addis Ababa, January, 2011.

When participants were asked if they were satisfied with the way sexual health services are advertised and delivered to street children, only 2.4% of respondents stated that they are satisfied with it and 21.8% of respondents reported as slightly satisfied and 75.8% of respondents were not satisfied with it (Figure 8).

The participants who were not satisfied tried to mentioned some of the reasons for service unsatisfaction. Lack children participation in program implementation and evaluation was the major reason 95 (29.7%) followed by poor advertisement of the service 94 (29.4%). while 71 (22.2%) mentioned lack of peer service and 27 (8.4) lack of confidentiality and poor distribution of condom and other contraceptive methods 33 (10.3%).

## 6. Results of Focus Group Discussion

The discussion was mainly focused on the risky sexual activity of street children, major sexual health problems of the street life, sexual and reproductive health services for street children, information of street children about STIs and HIV/AIDS, unwanted pregnancy, and service provider places. The discussion was started by asking the general question “*why they joined the street life.*” Almost all discussant from both sex mentioned the reasons that were listed in the quantitative part like searching jobs, conflict with family,poor family, and so forth. Female group mainly mentioned sexual related reasons like rape attempt, voluntary and involuntary unsafe sex resulting unwanted pregnancy. In connection with this, a 17-year-old girl FGD participant also stated the following:

*My aunt brought me from rural to Addis Ababa by convincing to attend school. I started life in Addis serving my aunts family and attending the class. But aunt's husband asked me many times for sex. I became feel bad when he come to home from work. One day when my aunt went to market, he came and tried to rape me. I swept from his hand and run away and never go back, start street life.*

Next to the general question about the reasons for streetism, discussants were invited to discuss commonly faced sexual and reproductive health problems. All participants were agreed that street girls are more exposed to sexual attacks and related problems than boys.

An 18-year-old male participant had the following to say in this regard;

*Stree boys only have the risk of contracting STIs including HIV/AIDS and sometimes sexually abused and psychological problems but female children have more than this. We chewed chat and had drink then no one could remember condom and have unsafe sex. In the morning we all concentrate on searching food. She remembers as she got pregnant when her abdomen gets larger or her menstruation stopped.*

This unsafe sexual relationship, among other factors, is believed to be the major source for the rapid increase of street mothers in Addis Ababa. As most of the participants mentioned, female street children are at most risk. They are vulnerable for unwanted pregnancy and STIs including HIV/AIDS. Sometimes they did not know when and from whom they had sex and got pregnant.

A 16-year-old street girl had the following to say:

*In the mid night there would be alcoholic boys and if they get female sleeping on the road, they will have even group and unusual sex.*

Sexual abuse and exploitation of male children is also one of the emerging social problems affecting the physical, social and psychological wellbeing of children in Addis Ababa. Almost all participants of the FGD session had heard at least once about this issue.

A 17-year-old street boy have said this:

*Male sexual abusing becomes common in Addis Ababa, especially around merkato distant bus station. It was last year summer, one 14 years old child was sent to shop to buy soap. Unfortunately the money was stolen and his mother told him to get out of the home. He came to the street and start to cry due hunger. In the mid time someone who is known thief and HIV carrier approach him and gave him biscuit and tea. Then he brought him to hidden place and had sex with him. We went there and fight with him. finally we brought him to police and he denied his activity and show them his card of HIV positive and they released him free. Legal action is not strong to punish those abusers.*

Another question was about their measures taken when sexual health problems were faced. As a result, diverse responses were given like report to police, abortion, give birth on the road and drop the baby on the road and so forth.

A 15-year-old girl says the following in this regard.

*I have no problem up to now, but I do not know what to do if I raped. Most probably I will kill myself.*

The next issue that was raised for discussion was about their general knowledge and information about sexual health services. They were asked about their experience (if any) with sexual health services in Addis Ababa. Both male and female discussants start the discussion by blaming the existing sexual and reproductive health services. They agree that they are totally disconnected from the existing service stream.

A 17-year-old male child suggests the following idea concerningthis issue.

*Governmental and nongovernmental organizations declared more as they did a lot on sexual heath to street children. But no free condom, no free contraceptive and no free treatment for us. I think now days everybody should have condom in his/her pocket. Most of the street children's attention is dominated by another issue like, cloth, food and shelter. So they won't have condom in their pocket but egger to have sex which leads them to unsafe sex. Sometimes we use chat plastic (yechat pestal) for sex. But I know it might have air, opening or easily ruptured, but we use it for confidence.*

Participants tried to mention some effective strategies to address the sexual and reproductive health needs of street children like mobile clinics and postal condom distribution.

An 18-years old state as following in this regard:

*There was postal style condom distribution in the areas where street children were congregated we insert fifty cent in it and have condom at the bottom. I'm in doubt about the presence of such services now. But it was effective in addressing social, physical and financial barriers of the service.*

Another 16-year-old street boy said the following about the mobile clinics:

*Mobile clinics give effective and easily accessible services for street children when avail. We can have condom whenever we want and we can check ourselves with the service without long waiting to get the services. But these mobile clinics seldom exist in the city. Most of the time mobile clinics were functional during the holiday and lasts maximum fifteen days. But no one wait the holidays to have condom.*

Barriers that prevent street children from using existing sexual health services were well discussed among participants. Both perceived and actual barriers were mentioned. Unaffordable cost is mentioned by many participants as a big barrier for utilization of the existing services among others. Lack of information about the services and the health facilities, ignorance of the consequence, unfriendly staff were mentioned as other barriers.

A 15-year-old boy stated the following in relation to staff character as service barrier.

*We do not have positive attitude for them. They give priority to the rich. They tried to judge the clients based on clothes them weare and physical status. We started to suffering from the gate keeper. They will never allow us to see the doctor. *

## 7. Results from Service Providers

Individual interview with service provider about staff characteristic, current practice, service quality, staff capacity, areas for improvement/change were conducted in three health facilities to present their ideas to one another and to make recommendations on how to make SRH services more appropriate and accommodating to street children. The service providers were selected based on the information obtained from Family Guidance Association Ethiopia area coordinator and other key informants about service providers.

In the interview program coordinators agreed that estimating the number of clients per day/week is difficult. This is because street children are not stable and will not attend based on appointment. But roughly coordinator of confidential clinic (piazza) stated that her organization sees about 15 street children per day and goal Ethiopia project coordinator estimates 50 street children per day but Sheger clinic coordinator express cannot estimate the number of street children per day and coordinator of Addis Ababa model clinic stated that they had no means of identification of street children from other clients. The types of sexual and reproductive health problems that street children were presented to the health facilities were discussed in the interview. As street children mentioned in the individual interview of the quantitative part, the program coordinators mentioned unwanted pregnancy, lack of awareness about family planning, STIs including HIV, and opportunistic infection are some of from others. Counselling, outreach reproductive health service, health education, clinical case management, VCT, distribution of condom were indicated by all of the interviewee as health services given from their project.

Program coordinator of confidential clinic, stated the following in relation to outreach services.

*The reality of providing such a service is that many street children clients will not attend arranged appointments and may disengage entirely for periods of time. In these stages the clients can be most vulnerable, and despite not looking for it, are often most in need of a sexual health service. It is therefore crucial to offer outreach and support to access sexual health services for those children who are most vulnerable and at risk.*

Research results indicate that sexual and reproductive health of street children is intricately connected to other aspects in their lives such as alcohol and other drug use, self esteem, and perception of judgment from peers. Trading sex is found to be a serious and growing problem in Addis Ababa for street children, particularly amongst street girls.

*Beside education and service provision, the basic thing is social and financial support. It must be to touch parallel; otherwise our intervention is not effective. Because children daily struggles to survive demanded that their sexual and reproductive health was a very low priority as a result they will back to street life for survival. We are unable to do this by now due to financial shortage. (In-depth interview, program coordinator, confidential clinic, Addis Ababa.)*

The next issue was about the types of SRH services that the project is unable to provide to the street children but should be provided. Different coordinators mentioned different SRH services that unable to provide to street children. VCT service (goal international), social and financial support (confidential clinic), and income generating skill (sheger clinic) are some of unaddressed SRH services for street children.

The main barriers/problems that service providers face in providing SRH services to street children were also raised and different barriers were mentioned. Among these problems, lack of training to build our capacity, problem in getting street children regularly and when needed, lack of resources,lack of coordination among service providers and policy restriction were some of the problems that negatively affect service provision.

In addition to the above issues, interviewees were also asked about the mechanism of promoting their SRH services to street children being confidential for the cases, taking time to discuss individually and in group, applying interactive teaching methods, making the environment friendly were some of techniques mentioned.

One of the questions that were raised *was have you taken any steps to make street children comfortable using the services and to create a street children-friendly environment?*. All of the respondents answered yes and tried to mention some of the steps they took. Coffee ceremony, panel discuusion, self support group (saving activities).

Regarding to the training received by the staff members of the organization, most of the interviewer said enough has not been done in this regard. Based on the responses given by the interviewer the main training given to the staff members was on child right, life skill, first aid, reproductive health and street life, friendly relationship with MARPs, drug abuse and its management (including alcohol and cigarette).

Interviews with coordinator of selected health facilities that exclusively provide sexual and reproductive health service to street children revealed that all programmes for street children, be they government run or supported by NGOs, lack adequate co-ordination between similar organizations. Almost all projects tried to address the same issue for street children independently which leads their effort less effective.

## 8. Discussion

Describing the street children's risky sexual behaviour (service need) and the existing programming response in Addis Ababa towards evolving recommendation for future programming was the major intention behind the present study. In seeking to address the issue of street children, it is essential to know why children are fledging in to the street of Addis Ababa. Many factors were listed for the reasons that push children to the street of Addis Ababa. Unemployment (28.9%), peer influence (21.8%), conflict with in the family (19.2%) and death of family (18.5%) were the reasons for joining the street life in this study. The reasons are similar with different percentage in the previous study. In one study done in Addis Ababa more than 41% of the respondent children joined street life because their families were poor to sustain them and more than a quarter (26.4%) of the respondent children was influenced by friends to leave home and 9.2% respondents mentioned conflict in the home as the reason behind leaving their homes [6]. The reasons have consistency for regions in the country. The result done in Dessie on street children showed that death of parents was reported by 36.2%, to look for a job by 23.0%, poor family by 12.8% and peer pressure by 4.4% [16].

Based on this study most participants (72.5%) were already sexually active, and 67.6% have had multiple sexual partners. Among sexually active children 90.4% were girls and 66.2% were boys. This result is slightly higher than the result obtained from the research among street children in Dessie town in which (67.9%) had ever practiced sexual intercourse [16]. This might be due to high sample size used in this study.

The mean and median ages of first sexual intercourse in this study were (15.4 and 15 years) for boys and (14.3 and 14 years) for girls. This result is also comparable with other studies in some part of the world. In Kinshasa, Democratic republic of Congo where the mean age of street children at first sexual intercourse was 14.3 years for males and 13.5 year for girls [26]. Another study done in India, showed the mean age of sexual intercourse for boys and girls were 15 and 13.2 years, respectively [11]. Here the main point is the proportion of sexually active in-school and out-of school children varied substantially by age. Out-of-school children who includes street children were more likely than their in-school counterparts to be sexually active in all age categories. For instance in one study in Ethiopia showed that 29 (3.1%) out-of-school children aged 10–14 years were sexually active, while there were no sexually active in-school children in the same age group. Similarly, the figures for sexually active out-of-school and in-school youth among the age groups 15–19 were 38.7% versus 84.8% [17]. Relatively comparable results were obtained in this study. 1.3% for age group of 10–12 years, 21.9% for 13–15 years age group and 76.8% for 16-16 age group have ever practice d sexual intercourse [17].

The reasons for early initiation of sexual intercourse among street children were also explained in this study. More than two-fifth (42.7%) of sexually active street children mentioned personal desire as the main reason for initiation of sex followed by love 34.4%. In another study personal desire was the dominant reason for initiation of sexual intercourse accounting about 38.2% followed by peer pressure 24.5% [18].

Children are addicted to chat, alcohol cigarette, ganja and shisha, even during the focus group discussion, some were chewing chat. Research results revealed that use of these substances influences sexual behavior in ways that increase the risk of acquisition of HIV and other STDs. The street child's decision on sexual behaviours such as whether to use a condom during sexual activity and whether to negotiate for sex or to use force (rape) depends on the level of intoxication. In general alcohol and other substance use often go along with the early sexual experiences, especially among boys. In this study, high proportion 284 (67.3%) of street children reported that they consumed some kind of substance. Chat was the dominant substance used by almost all 95.1% children. cigarette, ganja and shisha were reported as commonly used substance [19]. These substances were mentioned in other research results [6, 16]. Alcohol consumption is also common among street children. Almost two third (64%) of the respondents in this study were drinking alcohol. Among these alcoholic children, 50% drink sometimes (once per week) and 9.3% drink most of the time (three times per week) while 4.7% drink daily. Alcohol intake was investigated by other researchers and the result revealed high proportion of children drinking alcohol [8].

Living on the street, with no supervision, protection or guidance and wide risky sexual practice often makes street children vulnerable to a wide range of sexual and reproductive health problems. In total 198 (44.8%) among sexually active respondents have encountered sexual health problems. Among commonly mentioned sexual health problems, unprotected sex under the influence of chat/alcohol 60 (14.2%), unwanted pregnancy 40 (9.5%), rape attempt 35 (8.3%), STIs 24 (5.7%), rape 23 (5.5%) and abortion 7 (1.7%) (Table 7). Sexual health problems were more dangerous among females than males. Unwanted pregnancy in early age makes the problem among female street children is highly vulnerable than male counter parts [27]. Unintended pregnancy leads to a complicated process and it accounts for the majority of maternal mortality and morbidity [28]. Out of 108 female participants in this study, 66.6% had a history of unwanted pregnancy which is higher than the study in Dessie (25%) (16). out of the total unintended pregnancies in this study, 59.4% ends up with abortion which is almost similar to the same study in Dessie (55.5%) [16]. Reasons for Unintended pregnancy mentioned by participants were unavailability and misuse of contraceptives, slippery of condom; ignorance and rape were some of among others.

Despite the many sexual and reproductive health risks of street life, many Street children face multiple barriers to accessing sexual and reproductive health information and services. Among those who had sexual health information, the source from which the information was obtained is not accessible (mass media, 28.8%) and some of them are not reliable (friends, 19.4%). Only 16.8% reported health workers as their source of information. The responses from the study done in Dessie also showed that peers (55.4%), health workers (29.7%) and mass media (27.4%) were reported to be the major source of information on STIs, HIV/AIDS and unwanted pregnancy [16]. Majority of them do not know where to go for help when in trouble. In response to a question in this regard, (75.8%) of the participants indicated that they did not know where to go for help in case of sexual related problem. Only 24.2% of the participants have heard about such health facilities. The remaining 75.8% of interviewed children either have never heard (48.1%) or they are in doubt of the presence of such sexual health providers in Addis Ababa (27.7%). Low knowledge of street children about support giving organization is also observed in another research results. In one study only 36.7% said that they knew support giving organizations [6]. In Zambia, 47% of the sampled street children stated that they had nowhere to go in case they needed help with sexual related health problem [29]. This shows that less proportion of the total respondents were getting services from Support giving organizations and considering the fact that the highest number of these Organizations are working in Addis Ababa, the proportion of street children receiving services is extremely low.

In this study nearly one third (33.4%) of participants were incorporated in one organization for support at least once but they had left and come back to the streets. The reasons of rejoining the street life were non interest based services (27.0%) and unfriendly staffs (27.0%). While 21.3% of them stated limited services (provision of food only) as a reason for rejoining the street life.

Children's low awareness about the existing legal, medical and social support organization made them reluctant for action they are going to take when sexually abused or unwanted pregnancy faced. In this regard 30.0% of the respondent stated I will do nothing 24.9% and 11.0% responded that they will tell to their friend and 24.9% answered to report to police. Existing reporting practices were poor as investigated by other research in Addis Ababa. Accordingly slightly over half (51.8%) of the respondents kept the incident secret and they never attempted to disclose it to anyone. The remaining 48.2% reported the cases but the majority (42.4%) shared it to their intimate friends. Only (11.4%) reported it to legal enforcement bodies [6].

There are both perceived and actual barriers that prevent street children from using existing sexual and reproductive health services. Of the street children involved in this study, Only less than half 47.1% respondents reported ever visiting existing health institutions for SRH services. There was also considerable gender difference among those who visited institutions (63.2% females versus 37.5% males). among the reasons for nonuser the services, (53.7%). Unaffordable cost, long waiting time, inaccessible location, unfriendly staff, and inappropriate opening time were mentioned as the reason among non-visitors according to the percentage magnitude. These barriers were also indicated by respondents involved in another study in which services are too expensive (42%), too much waiting time (12.8%), feeling of embarrassment (12.2%), inconvenient health institutions (8.7%), too far health institutions (7.9%), poor handling and failure to keep privacy and confidentiality by health workers (7.6%) were some of the barriers [16].

Mobile health facilities which bring services directly to people are one method of addressing physical barriers to access for the most isolated and often the poorest populations like street children. Participant children in this study preferred separate SRH service delivery centre for street children (43.6%), mobile services (28.4%), separate room in existing heath setup (15.4%) and by trained street children (11.8%). This result is similar to the study done among the general young population in selected region of Ethiopia. The majority (over 72.0%) said that they would prefer to go to a separate health institution with a youth-friendly environment [30].

In the FGDs that were held with street children, most participants (both male and female) stated that they would prefer SRH services that were provided in specific health facilities organized for them in an accessible, convenient and confidential environment.

## 9. Strengths and Limitations of the Study

### 9.1. Strength

This research considers marginalized and neglected group of people about whom the information on sexual and reproductive health services is lacking.

The reliability of the data was maintained by predata collection training of the interviewers and the supervisors, close supervision by the principal investigator, and using pretested questionnaire.

Combining quantitative and qualitative data to triangulate the findings is strength of this study.

### 9.2. Limitations

There is no comprehensive baseline data available on the size of the street-child population in general and the sexual and reproductive health services in particular to street children. It is therefore difficult to estimate whether the situation is getting better or worse.

Recall bias cannot be ruled out, as the majority of children had dropped out-of-school at the primary level, leading to an overall lower education level in the group.

As in all self-reported behaviour studies, we also cannot rule out socially desirable answers to sensitive questions on sexual behaviour, which might have introduced biases of unknown magnitude and direction.

## 10. Conclusion and Recommendation

The most significant drivers of the street child population appear to be a complex of poverty, death of parents, conflict in family, job searching and limited alternatives.

Despite the efforts taken to address sexual and reproductive health issues among street children, research results showed that children face major sexual and reproductive health problems such as lack of information, unwanted pregnancy and unsafe abortion up to date.

The high proportion of street children in Addis Ababa is engaging in risky behaviors such as unprotected sex, forceful sexual intercourse and early initiation of drug abuse.

Street children in Addis Ababa need to be educated about ways to protect themselves from early pregnancy, STDs and HIV/AIDS.

There is demand of capacity building training for staffs of service provider.

It was observed that street children are poorly informed about sexual health and sexual health service provider organizations.

Friends and the media were obtained as the most important sources of information about sexuality and related problems.

Street children refuse to go to clinics because they do not think that clinics will serve them or too young and inexperienced to know how to find clinics.

Sex, alcohol consumption, life skill training and connectedness to NGOs were found to have statistically significant influence on sexual experience among street children.

Unaffordable cost, unfriendly staff and long waiting time were identified as major barriers in accessing SRH services for street children.

Service provider organizations are not advertising their services sufficiently for streetchildren.

Majority of street children had negative attitude towards service providers.

## 11. Policy Recommendation

### 11.1. Ethiopian Health Policy

The national health policy should recognize the need to develop and improve urban health extension services to meet the challenges of the street children.

In addition to this the policy should recognize street children are a population group with special needs.

The government guidelines should also exempt street children from health service user fee. Because based on this research result street children are vulnerable to sexual health problems, but underutilize the health services.

### 11.2. Child Development Policy

The Ethiopian child development policy should recognize street children as special target group and street children friendly strategies should be devised to reduce their vulnerability to sexually related problems. Currently the policy does not recognize street children as special target group requiring special attention.

### 11.3. Practical Actions to Support Street Children

To improve the access of health services to street children should not only provide free health care services, but also address the social, cultural and environmental factors that restrict access. Therefore efforts should be made to ensure that children who drop out-of-school and other street children are provided basic primary education. None formal or special primary education program will be critical in reaching children living on the street. In addition to this older street children (16–18 years) should be helped to secure properties and facilitated to acquire startup tools and capitals to undertake income generating activities.

### 11.4. Recommendations for Further Research

Greater understanding of the living condition and other health status of children living on the street is necessary to formulate appropriate policies, strategies, programs and health services for this highly vulnerable group. The current study covered only one city, Addis Ababa (four sub cities within the city). A large study covering the main cities in the country should be carried out. Such studies would provide more comprehensive understanding of the sexual and reproductive health status of street children in diverse city context. Further research is also needed to examine the attitude and practice of health personnel towards children living on the street.

## Supplementary Material

The supplementary materials include the study information sheet to be read for study participants and the structured questionnaire both in Amharic and English language. Semi-structured questionnaire for focus group discussion and key informant interview template for service providers also included in the supplementary materials.

## Figures and Tables

**Table 1:** Sociodemographic characteristics of street children in Addis Ababa, January, 2011 (*N* = 422).

Variables	Male		Female		Total	
	No	%	No	%	No	%
Type of street life						
On the street	48	15.3	65	60.2	113	26.8
Off the street	266	84.7	43	39.8	309	73.2
Sex	314	74.4	108	25.6	422	100
Age group (in years)						
10–12	15	4.8	4	3.7	19	4.5
13–15	100	31.8	28	25.9	128	30.3
16–18	199	63.4	76	70.4	275	65.2
Religion						
Orthodox Christian	176	56.1	46	42.6	222	52.6
Muslim	52	16.6	20	18.5	72	17.1
Protestant	57	18.2	39	36.1	96	22.7
Catholic	11	3.5	0	0	11	2.6
Has no religion	18	5.7	3	2.8	21	5.0
Ethnic group						
Amhara	102	32.5	27	25	129	30.6
Oromo	97	30.9	40	37	137	32.5
Tigry	52	16.6	23	21.3	75	17.9
Others	63	20.1	18	16.7	14	2.5
Marital status						
Single	285	90.8	63	58.3	348	82.5
Married	13	4.1	25	23.1	38	9.0
Divorced	16	5.1	20	18.5	36	8.5
Educational level						
Never attend	33	10.5	6	5.6	39	9.2
Read and write only	31	9.9	15	13.9	46	20.1
1–4 grades	98	31.2	44	40.7	142	53.8
5–8 grades	147	46.8	40	37.0	187	44.3
9–12 grades	5	1.6	3	2.8	8	19
Duration on the street						
	90	28.7	15	13.9	105	24.9
1–3 years	145	46.2	71	65.7	216	51.2
3–5 years	46	14.6	21	19.4	67	15.9
	33	10.5	1	0.9	34	8.1
Currently living with						
Peers	218	69.4	53	49.1	271	64.2
Alone	49	15.6	10	9.3	59	14.0
Boy/girlfriend	22	7.0	23	21.3	45	10.7
Parents	24	7.6	19	17.6	43	10.2
Others	1	0.3	3	2.8	4	0.9

**Table 2:** Socioeconomic characteristics of street children in Addis Ababa, January, 2011 (*N* = 422).

Variables	Number	Percent (%)
Income generating activities		
Carrying items	173	54. 1
Transferring message	31	7.3
Any occasional job	51	12.1
Commercial sex	49	11.6
Shoeshine	22	5.2
Car washing	34	8.1
Other	37	8.8
Average income per day		
<5 birr	18	4.5
5–10 birr	131	33.0
11–20 birr	146	36.8
21–50 birr	59	14.9
>50 birr	43	10.8
Ever helped by the organizations		
Yes	84	19.9
Yes but leaved now	141	33.4
No	197	46.7

**Table 3:** The distribution of street children by sex and their former residence in Addis Ababa, January, 2011 (*N* = 422).

Former residence	Sex		Total
	Male	Female	
In Addis Ababa			
Frequency	84	39	123
% within sex	26.8	36.1	29.1
Outside Addis Ababa			
Frequency	230	69	299
% within sex	73.2	63.9	70.9

Total			
Frequency	314	108	422
% within sex	100.0	100.0	100.0

**Table 4:** The types of substances or drugs consumed by the sample of drug consumers of street children in Addis Ababa, January, 2011.

Substances	Male		Female		Total	
	Yes	No	Yes	No	Yes	No
	No (%)	No (%)	No (%)	No (%)	No (%)	No (%)
Chat	196 (93.8)	13 (6.2)	74 (98.7)	1 (1.3)	270 (95.1)	14 (4.9)
Cigarette	170 (81.3)	39 (18.7)	45 (60.0)	30 (40)	215 (75.5)	69 (24.3)
Shisha	41 (19.6)	166 (79.4)	21 (28.0)	53 (70.7)	62 (21.8)	219 (77.1)
Benzene	17 (8.1)	187 (89.5)	0 (0.0)	73 (97.3)	17 (6.0)	260 (91.5)
Other	18 (8.6)	187 (89.5)	4 (5.3)	70 (93.3)	22 (7.7)	257 (90.3)

Total	442 (211.4)	592 (283.3)	144 (192)	227 (302.6)	586 (206.1)	819 (288.1)

**Table 5:** Alcohol consumption status among street children in Addis Ababa, January, 2011 (*N* = 422).

Variables	Male	Female	Total
	No (%)	No (%)	No (%)
Alcohol intake			
Never	112 (35.7)	40 (37.0)	152 (36.0)
Sometimes	161 (51.3)	50 (46.3)	211 (50.0)
Most of the time	28 (8.9)	11 (10.2)	39 (9.3)
Daily	13 (4.1)	7 (6.5)	20 (4.7)
Total	**422 (100)**	**108 (100)**	**422 (100)**
Sex after alcohol intake (*n* = 270)			
Yes	120 (59.4)	57 (83.8)	177 (65.6)
No	82 (40.6)	11 (16.2)	93 (34.4)
Total	**202 (100)**	**68 (100)**	**270 (100)**
Sex with condom after alcohol drink (*n* = 177)			
Yes	76 (63.3)	42 (73.7)	118 (66.7)
No	21 (17.5)	6 (10.5)	27 (15.3)
Do not remember	23 (19.2)	9 (15.8)	32 (18.0)
Total	**120 (100)**	**57 (100)**	**177 (100)**

**Table 6:** Sexual and reproductive health behaviors and practices of street children in Addis Ababa, January, 2011 (*N* = 422).

Variables	Male	Female	Total
	No (%)	No (%)	No (%)
Ever had sexual intercourse			
Yes	204 (65.0)	98 (90.7)	302 (71.6)
No	110 (35.0)	10 (9.3)	120 (28.4)
Reasons to have sex (*n* = 302)			
Exchange for money	0 (0)	25 (25.5)	25 (8.3)
Fall in love	54 (26.5)	50 (51.0)	104 (34.4)
Influence of khat/alcohol	7 (3.4)	0 (0)	7 (2.3)
Marriage	4 (2.0)	6 (6.1)	10 (3.3)
Peer pressure	13 (6.4)	8 (8.1)	21 (7.0)
Personal desire	125 (61.3)	4 (4.1)	129 (42.7)
Rape	1 (0.5)	5 (5.1)	6 (2.0)
Life time number of sexual partner (*n* = 302)			
One	32 (15.7)	14 (14.3)	46 (15.2)
Two and above	172 (84.3)	84 (85.7)	182 (84.8)
Sexual intercourse in the last 3 months (*n* = 302)			
Yes	112 (55.1)	85 (86.7)	197 (65.2)
No	92 (44.9)	13 (13.3)	105 (34.8)
Unwelcome sex in 12 months (*n* = 302)			
Yes	23 (11.3)	32 (32.7)	55 (18.2)
No	181 (88.7)	66 (67.3)	247 (81.8)
Risky activities for contracting HIV			
Sex without condom	62 (18.9)	39 (36.8)	101 (23.9)
Not remember	103 (32.9)	28 (26.4)	131 (31.0)
More than one sexual partner	31 (9.9)	27 (25.5)	58 (13.7)
Injury with sharp materials	64 (20.4)	4 (3.8)	68 (16.1)
Sex with commercial sex worker	33 (10.5)	0 (0.0)	33 (7.8)
Inconsistence condom use	12 (3.8)	5 (4.5)	17 (4.0)
Others	8 (2.6)	3 (2.8)	11 (2.6)
The most common SRH problem for street life			
Rape	68 (21.7)	39 (36.1)	107 (25.4)
STIs	63 (20.1)	17 (15.7)	80 (19.0)
Sexual exploitation	58 (18.5)	18 (16.7)	76 (18.0)
Lack of SRH information	55 (17.5)	16 (14.8)	71 (16.8)
Unwanted pregnancy	46 (14.6)	15 (13.9)	61 (14.5)
Lack of legal protection	24 (7.7)	3 (2.8)	27 (6.4)

**Table 7:** Sexual and reproductive health situation of female street children in Addis Ababa, January, 2011 (*N* = 108).

Variables	Number	Percent (%)
Ever been pregnant		
Yes	69	70.4
No	29	29.6
Life time number of pregnancy (*n* = 69)		
One	22	31.9
More than one	47	68.1
Pregnancies were wanted (*n* = 69)		
Yes	3	4.3
No	66	95.7
Reasons for pregnancy (*n* = 69)		
Rape	7	10.6
Failure of contraceptive	10	15.2
Ignorance	28	42.4
Unavailability of contraceptive	11	16.6
Other	10	15.2
Ever had child (*n* = 69)		
Yes	28	40.6
No	41	59.4
Undertake abortion (*n* = 69)		
Yes	59	85.5
No	10	14.5
Consulting before abortion (*n* = 59)		
Boyfriend	11	18.6
Peers	36	61.0
Health worker	12	20.4
Place of abortion (*n* = 59)		
Health center	34	57.6
Private clinic	6	10.2
Traditional abortionist	12	20.3
Self-induced	7	11.9

**Table 8:** Relationship between selected sociodemographic variables and sexual behavior of street children in Addis Ababa, January, 2011.

Variables	Ever had sexual intercourse		OR (95% CI)	
	Yes	No	Crude	Adjusted
Sex				
Male	204	110	0.19 [0.10, 0.38]∗∗	0.09 [0.03, 0.23]∗∗
Female	98	10	1.00	1.00
Had connection with NOGs				
Yes	36	48	1.00	1.00
No	254	84	2.6 (1.26, 5.18)∗	2.135 (1.25, 3.64)∗
Ever taken life skill training				
Yes	9	57	1.00	1.00
No	245	111	3.18 [1.15, 8.81]∗	2.87 [1.37, 6.00]∗
Length of street life				
<1 year	60	45	1.00	1.00
1–3 years	158	58	7.75 [2.23, 8.96]	5.96 [1.41, 7.22]∗∗
3–5 years	53	14	4.79 [1.12, 12.88]	4.29 [1.05, 17.59]∗
>5 years	31	3	4.56 [1.33, 20.02]	4.97 [1.04, 23.86]
Former residence				
Inside Addis	78	45	1.00	1.00
Outside Addis	224	75	1.9 [1.09, 3.51]∗	1.7 [1.10, 2.70]∗
Alcohol drinking				
Yes	235	85	6.84 [4.19, 11.18]∗∗	5.23 [3.73, 16.32]
No	67	35	1.00	1.00
Substance use				
Yes	230	54	3.9 [2.49, 6.10]∗∗	2.5 [1.42, 4.56]∗
No	72	66	1.00	1.00

NB ^*^ *P* < 0.05.

^**^ *P* < 0.001.

**Table 9:** Distribution of street children by their sexual health information status in Addis Ababa, January, 2011.

Variables	Male		Female		Total	
	No	%	No	%	No	%
Had information about HIV/AIDS, unwanted pregnancy (*n* = 422)						
Yes	134	42.7	57	52.8	191	45.3
No	180	57.3	51	47.2	231	54.7
Sources of information (*n* = 191)					
Mass media	37	27.6	18	31.6	55	28.8
Friends	28	20.9	9	15.8	37	19.4
School	17	12.7	4	7.0	21	11.0
Health workers	16	11.9	16	28.1	32	16.6
Informal talk	14	10.4	3	5.3	17	8.9
Street educator	14	10.4	2	3.5	16	8.5
Training	6	4.5	2	3.5	8	4.2
Posters	2	1.5	3	5.3	5	2.6
Reasons for lacking information (*n* = 231)						
No means of getting it	74	40.9	20	40.0	94	40.7
Less prioritized	63	34.8	16	32.0	79	34.2
It does not concern me	44	24.3	14	28.0	58	25.1

**Table 10:** Distribution of street children, who have information about HIV/AIDS and sexual related risks by age group in Addis Ababa, January, 2011.

Had an information about HIV and pregnancy	Age Group			Total
	10–12	13–15	16–18	
Yes				
Number	2	50	139	191
(%) Within age group	10.5	39.1	50.5	45.3
No				
Number	17	78	136	231
(%) Within age group	89.5	60.9	49.5	54.7

Total				
Number	19	128	275	422
(%) Within age group	100	100.0	100.0	100.0

**Table 11:** Knowledge of sexual and reproductive health service providers among street children who took life skill training in Addis Ababa, January, 2011.

Ever taken life skill training	Knowledge of SRH service provider			
	Yes	No	Do not know	Total
Yes	29 (43.9%)	20 (30.3%)	17 (25.8%)	66 (100%)
No	73 (20.5%)	183 (51.4%)	100 (28.1%)	356 (100%)

Total	102 (24.2%)	203 (48.1%)	117 (27.7%)	422 (100%)

**Table 12:** The distribution of street children by their knowledge of health centers that give sexual and reproductive health for them in Addis Ababa, January, 2011.

Knowledge of health centers that provide SRH services for street children	Sex		Total
	Male	Female	
Yes			
Number	63	39	102
% within sex	20.1%	36.1%	24.2%
No			
Number	155	48	203
% within sex	49.4%	44.4%	48.1%
Do not know			
Number	96	21	117
% within sex	30.6%	19.4%	27.7%

Total			
Number	314	108	422
% within sex	100%	100%	100%

**Table 13:** Street children's opinion about the sexual and reproductive health service characteristics in Addis Ababa, January, 2011.

Services character	Yes	No	Do not know	Total
Friendly	93	244	85	422
Not judgmental	72	264	86	422
Consider street culture	53	285	84	422
Good confidentiality	130	209	83	422
Found at appropriate location	47	285	85	422
Short waiting time	46	293	83	422

## Acronymes and Abbreviations

AIDS: Acquired immuno deficiency syndrome
CFSC: Consortium for street children
CRC: Convention for the right of children
EPIINFO: Epidemiological information
FGD: Focus group discussion
FSCE: Forum on street children in ethiopia
HIV: Human immuno deficiency virus
NGOs: Nongovernmental organizations
SPSS: Statistical package for social science
SRH: Sexual and reproductive health
STD: Sexually transmitted diseases
STIS: Sexually transmitted infections
UN: United Nation
UNICEF: United Nation International Children Emergency Fund
USAID: United States Agency for International Development
WHO: World Health Organization.
