# Supplementary material for: Assessment of Sexual and Reproductive Health Status of Street Children in Addis Ababa
Source: J Sex Transm Dis. 2013 Dec 26;2013:524076. doi: 10.1155/2013/524076 (PMC4437437; doi:10.1155/2013/524076)
Supplement: Supplementary file 1 — The supplementary materials include the study information sheet to be read for study participants and the structured questionnaire both in Amharic and English language. Semi-structured questionnaire for focus group discussion and key informant interview template for service providers also included in the supplementary materials. [file 524076.f1.docx]

***Supplementary materials***

## Study Information Sheet to be read for study participants

My name is _______________ I came from_____________________. I am a member of the research team of the Addis Ababa University,college of health science, school of public ealth

The following information is to inform you about a study we wish to conduct with you to ask your permission to participate in the study. The title for this study is **“*assessment of sexual and reproductive health status of street children in Addis Ababa”.*** We are interested in this area because sexual and reproductive health problems of street children are not given great attention yet it is an increasing issue. So if we better understand how governmental and nongovernmental organizations deliver sexual and reproductive health services to the street children, we can forward feasible and effective alternatives to address this ignored problem.

Participation in the research project is voluntary and you do not have to participate in the research project if you choose not to. In order to ensure anonymity, personal information will be coded with a number and stored in a locked office to which only those helping to collect data will have access. Any presentation or publication resulting from this study will not contain any identifiable information regarding you. Only those researchers assigned to this study will have access to the data.

This study has been reviewed and received approval from the Research Ethics Review Committee of Addis Ababa University, school of public health. Should you allow participating, you will have the option of withdrawing from the study at any time for any reason without consequence. Simply inform one of the data collectors that you wish to withdraw from the study and your information will be removed upon your request. As well, you have the right to not answer any question or to participate in any aspect of this project that you consider invasive, offensive or inappropriate.

If you have any questions or concerns about this request, please contact at 09-13-07-14-30 or

E-mail [hab2396HYPERLINK "mailto:hab2396@yahoo.com"@HYPERLINK "mailto:hab2396@yahoo.com"yahoo.com](mailto:hab2396@yahoo.com).

1. **Structured Questionnaire in English**

- Code No: ______________________
- Sub city: _______________________
- Site name: _____________________
- kebele (sefer name ):______________

| **Part I : Sociodemographic characteristics** | | | | | | | |
| --- | --- | --- | --- | --- | --- | --- | --- |
| **S.n** | | | **Questions** | **alternative answers** | | **Code** | |
| 101. | | | Sex | 1. Male 2. Female | | 1=male  2=female | |
| 102. | | | Age in years [enter number] | 1. ________years_________month   99. don’t know | | 99=don’t know | |
| 103. | | | Religion | 1. Orthodox 2. Muslim 3. Protestant 4. Catholic 5. No religion   88. Others, specify /_______________/ | | 88=other specify | |
| 104. | | | To which ethnic group do you belong? | 1. Amhara 2. Oromo 3. Tigray 4. guragie   88.Others,specify/________________/ | |  | |
| 105. | | | What is your current marital status? | 1. Currently married 2. Never married 3. Divorced 4. Widowed   88.Others,specify /_______________/ | |  | |
| 106. | | | Duration of street life [enter number] | 1. /_______/ years /________/ Months  99. Don’t know/remembe | |  | |
| 107. | | | What is the main reason to become to street? | 1. Peer pressure 2. Death of parents 3. Looking for a job 4. Due to alcoholic family 5. Lack of peace in the family 6. Displacement 7. change of life style 8. Others,specify /________________/   99. Don’t know/remember | |  | |
| 108. | | | Where is your former residence? | 1. Addis Ababa 2. Outside Addis Ababa | |  | |
| 109. | | | With whom do you live most of the time? | 1. Both parents 2. Mother 3. Father 4. Sister/brother 5. Friends/peers. 6. Boy / girl friend 7. Alone   88.Others specify /_______________/ | |  | |
| 110. | | | Where do sleep during the night? | 1. On the street 2. Small rented house 3. Plastic shelter 4. Families house   88.Others,specify/___________________/ | |  | |
| 111. | | | Education status | 1. Never attend school 2. Only read and write 3. 1 -4 grade 4. 5 – 8 grade 5. 9 -12 grade | |  | |
| 112. | | | Do you work to earn money for yourself? | 1. Yes 2. No | |  | |
| 113. | | | What do you do to earn money? [multiple answers are possible] | 1. Shoe shining 2. Carrying small items 3. Delivering messages 4. Attending and washing cars. 5. Exchange of money for sex   88.Others,specify /__________________/ | |  | |
| 114. | | | On average how much do you earn per day? | 1. Less than five birr 2. 5 -10 birr 3. 10 -20 birr 4. 30-50 birr 5. more than 50 birr | |  | |
| **PART-II: Concerning Substance and alcohol Abuse** | | | | | | | |
| 115. | | | Do you drink alcoholic beverage like Tela, Tej,  Beer, Arekie and the likes? | 1. Have never drunk 2. I have tried once or twice 3. I drink most of the time 4. I drink daily | |  | |
| 116. | | | If you drunk, have you ever had sexual intercourse after drinking? | 1. yes 2. No | |  | |
| 117. | | | If yes, have you used condom? | 1. yes 2. No   99. I don’t remember | |  | |
| 118. | | | Do you use drugs? | 1. Yes 2. No | |  | |
| 119. | | | Which drug do you use most of the time? | 1. Chat chewing 2. Ganja/Hashish, 3. cigarett smoking 4. Sniff Benzene 5. inject able drug   88.Other,specify /___________________/ | |  | |
| 120. | | | What initiates you to use drugs? | 1. Depression 2. Peer pressure 3. To protect hunger 4. To protect fear (sex,stell)   88.other,specify /__________________/ | |  | |
| **Part III: Concerning risky sexual and Reproductive health practices** | | | | | | | |
| 121. | | Have you ever had sexual intercourse? | | 1. Yes 2. No | |  | |
| 122. | | If yes, at what age did you first have sexual intercourse? (Enter number) | | 1. /________/ Age in years   99. Don't know/ remember | |  | |
| 123. | | Why did you decide to have sexual intercourse the first time? | | 1. I get married 2. Fell in love. 3. Personal desire. 4. Coercion (Rape). 5. To get money and other gifts. 6. Peer pressure 7. Influence of khat /alcohol   88.Others,specify /__________________/ | |  | |
| 124. | | With whom did you make your first sexual intercourse? | | 1. Husband/wife 2. with a steady boy/girl friend 3. with a casual boy/girl friend 4. with a family member 5. with commercial sex worker   88.others,specify /_______/ | |  | |
| 125. | | Have you had sexual intercourse within 3 monthes? | | 1. Yes 2. No | |  | |
| 126. | | How many sexual partners have you ever had? | | 1. /_______/ Number of partners 2. Don’t know / remember | |  | |
| 127. | | Have you been raped without the consent of you during the past 12 months? | | 1. Yes 2. No | |  | |
| 128. | | If yes, have you used condom or contraceptives? | | 1. Yes 2. No | |  | |
| 129. | | If no for Q No 128 why not? | | 1. it was unplanned 2. violetor oppossion 3. Too far to get condom or contraceptives(Not accessible) 4. Method was expensive 5. I have infrequent sex.   88. other ,specify /_________________/ | |  | |
| 130. | | Which activities that you performed might expossed you for HIV virus or unwanted pregnancy? | | 1. sexual intercourse without condom 2. multiple sexual intercourse 3. sexual intercourse withcomercial sex worker 4. cuting with sharp materials   99. don’t know/remember  88.other,specify/_________________/ | |  | |
| 131. | | What sexual and reproductive health problems you faced with in last 12 monthes? | | 1. unwanted pregnancy 2. abortion 3. alcohol infleunced unsafe sex 4. STIs 5. Coercion (Rape). 6. Coercion (Rape) attempte 7. No problem   88.other,specify /___________________/ | |  | |
| 132. | | Have you been pregnant? [***FOR FEMALES ONLY***] | | 1. Yes 2. No | |  | |
| 133. | | If yes, how many times have you been pregnant? (Enter number | | 1. /___________/ Times   99. Don't know/Remember | |  | |
| 134. | | Were all your pregnancies wanted? | | 1. Yes 2. No | |  | |
| 135. | | If no, how did you become pregnant? | | 1. Contraceptive method not available 2. Coercion (rape) 3. Method failure 4. lack of emphasis   88. Other, specify /__________/  99. Don’t know/ remember | |  | |
| 136. | | Did you give birth? | | 1. Yes 2. No | |  | |
| 137. | | Have you ever had abortion? | | 1. Yes 2. No | |  | |
| 138. | | If yes, how many times did you have abortion? | | 1. / _______/ Times   99. Don’t know/ remember | |  | |
| 139. | | For to have abortion, whom did you first discuss the issue with? | | 1. My boy friend 2. My peers 3. My parents 4. Health workers 5. Traditional healers 6. For an abortionist   88. Others, specify [______________] | |  | |
| 140. | | Where did you abort? | | 1. At public health institution 2. At private clinic 3. At abortionist’s house 4. I have induced it myself   88. Others, specify [_____________]  99. Don’t know/ remember | |  | |
| **Part IV:Concerning responses for sexual and reproductive health needs of street children** | | | | | | | |
| **4.1 sexual and reproductive health information and education** | | | | | | | |
| 141. | Do you have information about HIV/AIDS and unwanted pregnancy? | | | | 1. Yes 2. No |  | |
| 142. | If yes, where do you get this information? | | | | 1. Mass media (radio,TV…) 2. Training 3. Friends 4. Posters, pamphlets 5. Street health educators   88.Other,specify /_______________/ |  | |
| 143. | If your answer for Q No 141 is no reasons? | | | | 1. No alternative to get information 2. It does no conser me 3. Giving less emphasis b/c I have many priortised problems   88.Other,specify /_______________/ |  | |
| 144. | From whom would you like (trust) to receive information about sexual and reproductive health? | | | | 1. Mass media (radio,TV…) 2. training 3. friends 4. posters, pamphlets 5. street health educators   88. other /____________/ |  | |
| 145. | Have you ever attended special education on sexual and reproductive health for street children? | | | | 1. Yes 2. No   99. Don’t remember |  | |
| 146. | If the answer for Q No145 is yes, which organization prepared it? | | | | 1. Governmental 2. voluntary people 3. NGOs 4. religious organization   88. Other (specify) /__________/ |  | |
| 147. | In what form was the information delivered? | | | | 1. through dramatic form 2. Through invitation of famous persons 3. through street child participation 4. Through simple look and talk by health profession   88. other specify /_______________/ |  | |
| 148 | If you are not comfortable with the way they deliver the information, in what form would you like? | | | | 1. dramatic 2. peer teaching 3. famous people 4. former street children   88.Other /_____________/ |  | |
| **4.2 life skill development practices** | | | | | | | |
| 149 | Have you helped by organizations/ | | | | 1. yes 2. no 3. yes but laved now |  | |
| 150 | If yes, which item was givn to you? | | | | 1. food 2. shelter 3. health 4. education 5. wood/metal work   88.other /__________/ |  | |
| 151 | If you are leaved the organization now why? | | | | 1. unfriendly staff approach 2. limited services 3. the service was not based on our need 4. the organization is not unctional   88. other |  | |
| 152 | What is your future plan to be if things are suitable? | | | | 1. merchant 2. doctor 3. teacher 4. nothing   88.other /_______________/ |  | |
| 153 | Which activities can be done by yourself? | | | | 1. how to use condom 2. how to protect myself from rape 3. when and how to use contraceptive 4. different games including football 5. carwashing and other jobs   88.other /___________________/ |  | |
| 154 | How did you spent most of your day times | | | | 1. sleeping 2. walkingg 3. chewing 4. begging 5. playing foot ball   88.other/______________/ |  | |
| 155 | Have you taken any life skill training? | | | | 1. yess 2. no |  | |
| 156 | If no, what is the reason? | | | | 1. has no interst 2. noot understand the benefit 3. lack of opprtunity 4. lack of traing fee   88. other /__________________/ |  | |
| 157 | What is your current interst to take trainig? | | | | 1. metal/wood work 2. food preparation 3. shoshine   88. other |  | |
| **4.3 Sexual and reproductive health services** | | | | | | | |
| 158 | Where do you go for advices related to SRH and substance use? | | | | 1. Health center 2. Religious leaders 3. Close friends who is not use substance 4. NGOs working on street children   88. Other (specify) /___________/ |  | |
| 159 | Why you prefere the above place? | | | | 1. good confidentiality 2. they easily understand me 3. short waiting time 4. affordable cost   88.other /_______________/ |  | |
| 160 | from your or your friends’ experience,what is the main SRH proble | | | | 1. rape 2. sexual eploitation 3. lack of legal protection 4. STIs 5. unwanted pregnancy 6. lack of SRH nformaton   88. other/__________/ |  | |
| 161 | Is there any organization in your location that provid condom or other contraceptive for street children? | | | | 1. yes 2. no   99.don’t know |  | |
| 162 | if yes,mention the type of organization | | | | 1. public hospital 2. public clinic 3. private hospital 4. private clinic 5. family guidance 6. NGO clinic   88. other/____________/ |  | |
| 163 | Have ever visited these health service providers for SRH service? | | | | 1. yes 2. no |  | |
| 164 | if no,what was the reason? | | | | 1. unfriendly staff 2. unaffordable cost 3. inappropriate location 4. inappropriate oppening ttime 5. long waiting time   88.other /____________/ |  | |
| 165 | if your aswer for Q No 163 is yes,what are the main SRH services given by the organizations | | | | 1. condom/contraceptive provision 2. ART service 3. VCT service 4. FP education and service   88. other/___________/ |  | |
| 166 | Which SRH ervice given to street children free of cost | | | | 1. condom/contraceptive 2. VCT service 3. education about FP and available methods 4. education about HIV transmission 5. nothing is free   88.other /_______________/ |  | |
| 167 | If you are going to faced rape or sexual exploitaion, what measure wii you take? | | | | 1. report to police 2. visite the doctor 3. tell to friends for help 4. nothing   88. other /______________/ |  | |
| 168 | Have you been confused where to go when you in need of SRH services? | | | | 1. yess 2. yes,more than once 3. no   99.don’t know/ remember |  | |
| 169 | If yes, what type of SRH service did you need? | | | | 1. Test for STIs including HIV 2. counselling for pre and post test 3. care and help for HIV problem 4. condom and other contraceptiv 5. legal abortion 6. delivery service   88.other/_____________/ |  | |
| 170 | What was your or your friends’ experience of using any of local sexual and reproductive health services? (thick) | | | | 1. Friendly 2. No-judgmental 3. Understand culture of street children 4. Confidentiality 5. apropriate location 6. Opened every time 7. short waiting time | **Yes** | **No** |
|  |  |  |  |  |  |  |  |
| 171 | Which of the following strategies  is /are implemented by  Organizations to address your sexual and reproductive health problems? | | | | 1. Increase involvement of street children in sexuality education 2. Increase focus on prevention education, in addition to abstinence-focused education 3. Increase access to reproductive health services for street children 4. Focus on both reducing risk factors and strengthening protective factors   88. Other (list) /_______________/ |  | |
| 172 | what are the main barriers for street children to have SRH services? | | | | 1. lack of knowledge about the avialable services 2. lack of knowledge about the consequences 3. stigma and discremination 4. unaffordable cost 5. unfriendly staff 6. distant location   88. other/___________/ |  | |
| 173 | in what way the SRH should be given to street children inorder to ensure that the service is effective? | | | | 1. in separate facility for street children 2. in separate room in existinfacilities 3. mobile services 4. through trained street children |  | |
| 174 | To ensure the higest number condom user,in what way the condom should be distributed to street childrem? | | | | 1. free in public phone 2. by coopriating with private pharmacies 3. by trainig street children mobile service 4. using local bars and restourants   88.otther/______________/ |  | |
| 175 | Is the current SRH services satsfactory for street children? | | | | 1. yes 2. slightly yes 3. absolutly no |  | |
| 176 | if your answer for Q No 175 is no what is thereason? | | | | 1. the service is poorly advertised 2. no peer service program 3. children are not participating in planninig,implementing and evaluating activities 4. lack of confidentiality 5. problems on condom provision   88. other/__________________/ |  | |

**Thank You!!**

##

## *Structured Questionnaire In Amharic*

**የቃለ መጠይቅ ቅፅ**

በአዲስ አበባ ዩኒቨርሲቲ የህብረተሰብ ጤና ት/ክፍል የወሲባዊና ሥነተዋልዶ ጤና ፍላጎትና በዘርፉ የሚሰሩ መንግስታዊና መንግስታዊ ያልሆኑ ድርጅቶች እየሰጡት ያለውን ምላሽ አስመልክቶ በጎዳና ተዳዳሪ ልጆች ላይ ለሚደረግ ጥናት መረጀ ለማሰባሰብ የተዘጋጀ ቃል መጠየቅ ቅፅ፤

ጤና ይስጥልን፡፡ ስሜ_________________ይባላል የመጣሁት ከ________________. በአዲስ አበባ ዩኒቨርሲቲ የህብረተሰብ ጤና ት/ት ክፍል የጥናት ቡድን አባል ነኝ፡፡የዚህ ጥናት ዋና አላማ እድሜቸው ከ 18 ዓመት በታች እድሚ ባላቸው የጎዳና ተዳዳሪ ልጆች ላይ የወሲባዊና የሠነ- ተዋልዶ ጤናን አስመልክቶ መረጃ ለማሰባሰብ ነው። ከአንተ/ቺ እድሜ ክልል ካሉ አቻ ጓደኞችህ/ሸ መካከል የዚህ ጥናት ተሳታፊ ለመሆን እድል አግኝተሃል/ሻል ጥናቱ በቃለ ምልልስ የሚከናወን ነው። ጥቂት ጊዜህን/ሽን መስዋት በማድረግ ለጥናቱ የበኩልህን/ሽን እድታደርግ/ጊ እንጠይቃለን፡፡ ከአንተ/ቺ የምናገኘው መረጃ በቀጣይ የተሸለ የጎዳና ተዳዳሪ ልጆች የሰነተዋልዶ እና ወሲባዊ ጤና አገልግሎት ለመስጠት እንደሚረዳ ይታመንበታል፡፡

ውይይቱ የግል ህይወትንና ሚስጥርን ስለሚያካትት በቃለ ምልልሱ ወቅት አንተ/ቺ እና እኔ ለብቻችን ሆነን ልንነጋገርበት የምንችልበት የተለየ ቦታ በናገኝ እጅግ ይመረጣል፡፡ ይህ አንተ/ችና እኔ የምናደረገው ውይይት እስከ መጨረሻው በሚስጥር የሚያዝ መሆኑን ላረጋግጥልህ/ሽ እወዳለሁ፡፡ ስለዚህ ይረዳ ዘንድ ማንኛውም የመትሰጠው /ጪው መረጃ ቁጥር እንዲሰጠው ከመደረጉ ሌላ ስምህ /ሽ በዚህ ቅፅ ላይ አይሞላም፡፤ በተጨማሪም የምተሰጠውን/ ጭውን መረጃ በየትኛውም መንገድ ለማንም ሰው አሳልፎ አይሰጥም፡፡

የትኛውም ከጥናቱ የተያየዘ ሪፖርት አንተን/ችን ላይቶች ሊያሳውቆ አይችልም፡፡ ቃለ ምልልሱ ሙሉ በሙሉ በፍቀደኝነት ለይ የተመሰረተ ነው፡፡

በጥናቱ ለመሳተፉ ፈቃደኛ ነህ/ሽ? --------- አዎ ----- አይደለሁም

የጠያቂው ስም ------------------------

ቃለ መጠይቁ የተደረገበት ቀን --------- / ---------- 2003 ፊርማ -------

የተቆጣጣሪው ስም --------

ፊርማ ----------- ቀን ----------/------2003

ቃለመጠይቁ የወሰደው ሰዓት ----------------

***የስነ-ተዋልዶና ወሲባዊ ጤና ፍላጎትን ተመሰርቶ ለጎዳና ተዳዳሪ ልጆች እየተሰጠ ያለውን ድርጅታዊ ምላሽ አስመልክቶ መረጃ ለማሰባሰብ የተዘጋጀ ቃለ መጠይቅ፡፡***

- *የመጠይቁ መለያ ቁጥር __________________*
- *ክፍለ ከተማ ___________________*
- *የቀበሌው ስም _________________*
- *የቦታው ስም ___________________*
- *መጠይቁ የተደረገበት ሰዓት_________________*

| *ክፍል አንድ ፡- አጠቃላይ የግለሰብ መረጃ* | | | | | | | | | | |  |  |  |  |  |  |  |  |
| --- | --- | --- | --- | --- | --- | --- | --- | --- | --- | --- | --- | --- | --- | --- | --- | --- | --- | --- |
| *ተ.ቁ* | | *ጥያቄ* | *አማራጭ መልሶች* | | | | | *ኮድ* | | |  |  |  |  |  |  |  |  |
| *101* | | *ፆታ* | 1. *ወንድ* 2. *ሴት* | | | | | *1=ወንድ*  *2=ሴት* | | |  |  |  |  |  |  |  |  |
| 102 | | *እድሜ (በአመት)* | 1. *____ዓመት_____ወር*   *99. አላውቅም/አላስታውስም* | | | | | *99=አላውቅም/አላስታውስም* | | |  |  |  |  |  |  |  |  |
| 103 | | *ሃይማኖት* | 1. *ኦርቶዶክስ ክርስቲያን* 2. *ሙስሊም* 3. *ፕሮተስታንት* 4. *ካቶሊክ* 5. *ሀይማኖት የለኝም*   *88. ሌላ ካለ ይጠቀስ / /* | | | | | *88=ሌላ ካለ ይጠቀስ* | | |  |  |  |  |  |  |  |  |
| 104 | | *የየትኛው ብሔር አባል ነህ?* | 1. *አማራ* 2. *ኦሮሞ* 3. *ትግሬ* 4. *ጉራጌ* 5. *ሌላ ካለ ይጠቀስ / /* | | | | |  | | |  |  |  |  |  |  |  |  |
| 105 | | *የትዳር ሁኔታ (በአሁኑ ወቅት)?* | 1. *አግብቻለሁ* 2. *አላገባሁም* 3. *ተፋትቻለሁ* 4. *ባሌ/ሚስቴ ሞታብኛለች*   *88. ሌላ ካለ ይጠቀስ / /* | | | | |  | | |  |  |  |  |  |  |  |  |
| 106 | | *ጎዳና ላይ ከመጣህ /ሽ ስንት ጊዜሽ/ህ ነው?* | 1. *______ ዓመት ________ወር*   *99. አላውቅም/አላስታውስም* | | | | |  | | |  |  |  |  |  |  |  |  |
| 107 | | *ወደ ጎዳ የወጣህበት/ሽበት ምክኒየት ምን ነበር?* | 1. *በጓደኛ ግፊት* 2. *ወላጆች ስለምቱ* 3. *ሰራፍለጋ* 4. *ወላጆቸ የመጠጥ ሱሰኛ መሆን* 5. *በቤተሰብ ውስጥ ስምምነት ስለሌለ* 6. *በተፈናቀሎ* 7. *የአኗኗር ዘይቤ ለውጥ*   *88. ሌላ ካለ ይጠቀስ / /* | | | | |  | | |  |  |  |  |  |  |  |  |
| 108 | | *ከዚህ በፊት የት ነበር የምትኖረው?* | 1. *አዲስ አበባ* 2. *ከአዲስ አበባ ውጭ ቦታው ይጠቀስ* | | | | |  | | |  |  |  |  |  |  |  |  |
| 109 | | *አብዛኛውን ጊዜ ከማን ጋር ነው የምትኖረው/ሪው?* | 1. *ከሁሉም ወላጆች ጋር* 2. *ከእናቴ ጋር* 3. *ከአባቴ ጋር* 4. *ከእህቴ /ወንድሞቼ ጋር* 5. *ጓደኞቼ ጋር* 6. *ከሴት (ወንድ) ጓደኛየ ጋር* 7. *ብቻየን*   *88. ሌላ ካለ ይገለጽ / /* | | | | |  | | |  |  |  |  |  |  |  |  |
| 110 | | *ሌሊቱን የት ታሳልፋለህ/ሽ?* | 1. *ጎዳና ላይ* 2. *ትንሽ ኪራይ ቤት* 3. *ፐላስቲክ መጠለያ ውስጥ* 4. *የቤተሰብ ቤት ውስጥ*   *88. ሌላ ካለ ይጠቀስ / /* | | | | |  | | |  |  |  |  |  |  |  |  |
| 111 | | *የትምህርት ሁኔታ* | 1. *ትምህርት ቤት አልገባሁም* 2. *ማንበብና መጻፍ ብቻ እችላለሁ* 3. *1-4 ክፍል* 4. *5-8 ክፍል* 5. *9-12 ክፍል*   *88. ሌላ ካለ ይገለጽ/ /* | | | | |  | | |  |  |  |  |  |  |  |  |
| 112 | | *ገንዘብ ለማግኘት ስራ ትሰራለህ/ሽ?* | 1. *አዎ* 2. *አልሰራም ወደ ተ.ቁ 115* | | | | |  | | |  |  |  |  |  |  |  |  |
| 113 | | *ስራ የምትሰራ/ሪ ከሆነ ምን ዓይነት ስራ ነው የምትሰራው/ሪው?*  *(ከአንድ በላይ መልስ ይቻላል)* | 1. *ጫማ መጥረግ* 2. *እቃ መሸከም* 3. *መላላክ* 4. *መኪና ማጠብ* 5. *የወሲብ ንግድ*   *88. ሌላ ካለ ይጠቀስ / /* | | | | |  | | |  |  |  |  |  |  |  |  |
| 114 | | *በአማካኝ በቀን ስንት ታገኛለህ/ሽ ?* | 1. *ከ 5 ብር በታች* 2. *ከ 5 - 10 ብር* 3. *ከ10 - 20 ብር* 4. *ከ 30- 50 ብር* 5. *ከ 50 ብር በላይ* | | | | |  | | |  |  |  |  |  |  |  |  |
| ***ክፍል ሁለት፡ አደንዛዥ ዕፅንና የአልኮል መጠጥን በተመለከተ*** | | | | | | | | | | |  |  |  |  |  |  |  |  |
| 115 | | *እንደ አረቄ ጠላ እና ጠጅ የመሳሰሉትን የአልኮል መጠጦች ትጠጣለህ/ሽ ?* | | 1. *ጠጥቼ አላውቅም - ወደ ጥ.ቁ 119* 2. *አልፎ አልፎ አጠጣለሁ* 3. *ብዙውን ጊዜ እጠጣለሁ* 4. *በየቀኑ እጠጣለሁ* | | |  | | | |  |  |  |  |  |  |  |  |
| 116 | | *የአልኮል መጠጦችን የምትጠጣ/ጭ ከሆነ ሰክረህ/ሽ ታውቃለህ/ሽ?* | | 1. *ሰክሬ አላውቅም* 2. *አንድ ሁለቴ ሰክሬያለሁ* 3. *በጠጣሁ ቀን ሁሉ እሰክራለሁ*   *88. ሌላ ካለ ይጠቀስ* | | |  | | | |  |  |  |  |  |  |  |  |
| 117 | | *ሰክረህ/ሽ የምታውቅ/ቂ ከሆነ ሰክረህ/ሽ 118ወሲብ ፈጽመህ/ሽ ታውቃለህ/ሽ?* | | 1. *አወ* 2. *ፈጽሜ አላውቅም* | | |  | | | |  |  |  |  |  |  |  |  |
| 118 | | *ከላይ መልስህ/ሽ አወ ከሆነ ኮነዶም ተጠቅመሀል/ሻል?* | | 1. *አወ* 2. *አልተጠቀምኩም*   *99. አላሰታውስም* | | |  | | | |  |  |  |  |  |  |  |  |
| 119 | | *የአነቃቂ/አደንዛዥ ዕፅ ተጠቃሚ ነህ/ሽ?* | | 1. *አዎ* 2. *አይደለሁም* ***ወደ ጥያቄ ቁ. 122*** | | |  | | | |  |  |  |  |  |  |  |  |
| 120 | | *መልስህ/ሽ አወ ከሆነ ከምትጠቀማቸው አነቃቂ /አደንዛዥ ዕፆች ውስጥ በብዛት የምትጠቀመው የትኛውን ነው?* | | 1. *ጫት* 2. *ጋንጃ /ሃሽሽ* 3. *ሲጋራ* 4. *ቤንዚን* 5. *በመርፌ የሚወሰዱ ዕፆች*   *88. ሌላ ካለ ይጠቀስ/_____________/* | | |  | | | |  |  |  |  |  |  |  |  |
| 121 | | *እነዚህን አነቃቂ/አደንዛዥ ዕፆች ለመጠቀም የሚያነሳስህ ነገር ምንድ ነው ?* | | 1. *ድብርትን ለማሰወገድ* 2. *በጓደኛ ግፊት* 3. *ረሃብን ለመከላከል/ለመርሳት* 4. *ፍርሃትን ለመከላከል (ለሴክስ፣ ለስርቆት)*   *88. ሌላ ካለ ይጠቀስ / /* | | |  | | | |  |  |  |  |  |  |  |  |
| ***ክፍል ሦሰት፡ አደገኛ ወሲባዊና ስነ- ተዋልዶዊ ተግባሮችን በተመለከተ*** | | | | | | | | | | |  |  |  |  |  |  |  |  |
| 122 | | *የግብረ ስጋ ግንኙነት ፈፅመህ ታውቃለህ?* | | | 1. *አዎ* 2. *ፈፅሜ አላውቅም* | |  | | | |  |  |  |  |  |  |  |  |
| 123 | | *ለጥያቄ 122 መልስዎ አዎ ከሆነ በምን ያህል እድሜህ/ሽ ነበር የግበረስጋ ግንኙነት የፈጸምከው/ሽው* | | | 1. *በ----------------አመቴ*   *99. አላውቅም /አላስታውስም* | |  | | | |  |  |  |  |  |  |  |  |
| 124 | | *የግብረ ስጋ ግንኙነት ለመፈፀም ውሳኔ ላይ የደረስከው/ሽው ለምን ነበር?* | | | 1. *በጋብቻ ምክኒያት* 2. *በፍቅር ምክንያት* 3. *የግል ፍላጎት* 4. *በመደፈር* 5. *ገንዘብ ለማግኘት* 6. *በጓደኛ ግፊት* 7. *በጫት /አልከል ግፊት*   *88. ሌላ ካለ ይጥቀሱ / /*  *99. አላስታውስም* | |  | | | |  |  |  |  |  |  |  |  |
| 125 | | *ባለፉት 3 ወራት ወስጥ ወሲብ ፈፅመህ/ሽ ታውቃለህ/ሽ* | | | 1. *አወ (ከማን ጋር------------------------------)* 2. *ፈፅሜ አላውቅም* | |  | | | |  |  |  |  |  |  |  |  |
| 126 | | *ለመጀመሪያ ጊዜ ከማን ጋር ነበር የግብረስጋ ግንኙነት የፈጸምከው/ሽው?* | | | 1. *ባል/ሚስት ጋር* 2. *የፍቅር ጓደኛ ጋር* 3. *ባጋጣሚ ካገኘሁት /ሴት/ወንድ ጋር* 4. *ከቤተሰብ አባላት ጋር* 5. *ከሴተኛ አዳሪ ጋር*   *88. ሌላ ካለ ይጠቀስ / /*  *99. አላውቅም/አላስተውስም* | |  | | | |  |  |  |  |  |  |  |  |
| 127 | | *ከምን ያህል ሰዎች ጋር የግብረ ስጋ ግንኙነት ፈጽመሀል/ሻል?* | | | 1. *------------ ሰዎች ጋር*   *99. አላውቅም/አላስታውስም* | |  | | | |  |  |  |  |  |  |  |  |
| *128* | | *ባለፍት 12 ወራት ውስጥ ከፈቃድህ/ውጭ ተገደህ/ሽ የግብር ስጋግንኙነት ፈፅመሀል/ሻል?* | | | 1. *አዎ* 2. *አልፈፀምኩም* | |  | | | |  |  |  |  |  |  |  |  |
| 129 | | *ለጥያቄ ቁጥር 128 መልስህ/ሽ አዎ ከሆነ የወሊድ መካላከያ ወይ የአባላዘር በሽታ መከላከያ ተጠቀመሀል/ሻል?* | | | 1. *አዎ ( ጥቅም ላይ የዋለው መከላከያ ይጠቀስ)* 2. *አልተጠቀምኩም* | |  | | | |  |  |  |  |  |  |  |  |
| 130 | | *ለጥያቂ ቁጥር 128 አልተጠቀምኩም ከሆነ፣ያልተጠቀምክበት/ሽበትምክኒያትምንድንነው?* | | | 1. *በጀላይ አልነበረኝም* 2. *በአካባቢው ማግኘት ስለማልችል* 3. *ያስገደደኝ አካል ፍቃደኛ አልነበረም* 4. *በቸልተኝነት* 5. *ድንገተኛ ስለነበር* 6. *ሌላ ካል ይጠቀስ / /* | |  | | | |  |  |  |  |  |  |  |  |
| 131 | | *ካሁን በፊት ለኤች አይ ቪ ቫይረስ ወይም ላልተፈለገ እርግዝና የሚጋልጥ ተግባር ፈፅሚያለሁ ብለህ/ሽ የምትገምተው የቱን ነው? (ከአንድ በላይ መልስ ይቻላል)* | | | 1. *ያለኮነዶም የፈፀምኩት የግብረ-ስጋ ግንኙነት* 2. *ካአንድ በላይ የወሲብ ጎደኛ ስለነበረኝ* 3. *ከሴተኛ አዳሪ ጋር ወሲብ ስለፈፀምኩ* 4. *ስለታም በሆኑ ነገሮች ጉዳት ደርሶብኝ ስለነበር* 5. *ሁልጊዜ ኮነዶም ስለማልጠቀም*   *88. ሌላ ካለ ይጠቀስ / /*  *99. አላውቅም/አላስታውስም* | |  | | | |  |  |  |  |  |  |  |  |
| 132 | | *ባለፍት 12 ወራት ውስጥ ያጋጠመህ ወሲባዊ ወይም ስነተዋለዶዊ የጤና ችግር ምን ነበር?* | | | 1. *ያልተፈለገ እርግዝና* 2. *ፅንስ የማስወረድ ተግባር* 3. *በስካር ግፊት ልቅ ግብረ ስጋ ግንኙነት* 4. *የአባላዘር በሽታ* 5. *ተገዶ የመደፈር ችግር* 6. *ተገዶ የመደፈር ሙከራ* 7. *ያጋጠመኝ ችግር የለም*   *88. ሌላ ካለ ይጠቀስ / /* | |  | | | |  |  |  |  |  |  |  |  |
| 133 | | *እርግዝና አጋጥሞሽ ያውቃል? ( ለሴቶች ብቻ)* | | | 1. *አዎ* 2. *አላውቅም* ***ወደ ጥ .ቁ .142*** | |  | | | |  |  |  |  |  |  |  |  |
| 134 | | *ከላይ መልስሽ አዎ ከሆነ ለምን ያህል ጊዜ ነበር እርግዝና የጋጠመሽ (ቁጥር ይገለፅ)* | | | *1. --------- ጊዜ*  *99. አላውቀም /አላስታውስም* | |  | | | |  |  |  |  |  |  |  |  |
| 135 | | *ሁሉም እርግዝና ክስተቶች የሚፈለጉ ነበሩ?* | | | 1. *አዎ* 2. *አይፈለግም ነበር* | |  | | | |  |  |  |  |  |  |  |  |
| 136 | | *ከላይ መልስሽ አይፈለጉም ነበር ከሆነ እንዴት ልታረግዢ ቻልሽ* | | | 1. *የእርግዝና መከላከያ አቅርቦት ማጣት* 2. *አስገድዶ በመደፈር* 3. *የእርግዝና መከላከያው አለመሰራቱ* 4. *አላስበኩበትም ነበር*   *88. ሌላ ካለ ይጠቀስ / /*  *99. አላውቀም /አላስታውስም* | |  | | | |  |  |  |  |  |  |  |  |
| 137 | | *ልጅ ወልደሽ ታውቂያለሽ* | | | 1. *አዎ* 2. *አላውቅም* | |  | | | |  |  |  |  |  |  |  |  |
| 138 | | *የፅንሽ የማቋረጥ ተግባር ፈፀመሽ ታውቂያለሽ?* | | | 1. *አዎ* 2. *አላውቅም* | |  | | | |  |  |  |  |  |  |  |  |
| 139 | | *መልስሽ አዎ ከሆነ ለምን ያህል ጊዜ አቋርጠሻል?* | | | 1. *------ ጊዜ*   *99. አላውቀም/አላስታውስም* | |  | | | |  |  |  |  |  |  |  |  |
| 140 | | *ፅንሱን ከማቋረጥሽ በፊት ከማን ጋር በጉዳዩ ላይ መከርሽ?* | | | 1. *ከወንድ ጓደኛዬ ጋር* 2. *ከአቻ ጓደኞቼ ጋር* 3. *ከቤተሰቦቼ ጋር* 4. *ከጤና ባለሙዎች ጋር* 5. *ከልምድ አዋላጆች ጋር* 6. *ማስወረድ ስራ ከሚሰሩ ባለሙያዎች ጋር*   *88. ሌላ ካለ ይጠቀስ / /* | |  | | | |  |  |  |  |  |  |  |  |
| 141 | | *ፅንሱን ያቋረጥሽው የት ቦታ ነበር?* | | | 1. *ከጤና ተቋም ውስጥ* 2. *የግል ክሊነክ* 3. *ከባህላዊ አዋላጆች* 4. *በራሴ የተለያዩ መደሃኒቶችን በመውሰድ*   *88. ሌላ ካለ ይጠቀስ / /*  *99. አላውቅም /አላስታወስም* | |  | | | |  |  |  |  |  |  |  |  |
| *ከፍል አራት፡-ወሲባዊናሥነ- ተዋልዶ ጤናን ለማበልጸግ ለጎዳና ልጆች እየተደረጉ ያሉትን ተግባራት ለመዳሰስ* | | | | | | | | | | |  |  |  |  |  |  |  |  |
| - 1. ***የስነ-ተዋልዶና ወሲባዊ ጤና መረጃና ትምህርትን በተመለከተ*** | | | | | | | | | | |  |  |  |  |  |  |  |  |
| 142 | *ኤች አይ ቪ ኤድስ እና ያልተፈለገ እርግዝናን እንዲሁም ሌሎች ከወሲባዊ ጤና ጋር ተያያዥነት ስላላቸውነገሮች መረጃ አለህ/ሽ?* | | | | 1. *አዎ* 2. *የለኝም* | | | | | |  |  |  |  |  |  |  |  |
| 143 | *ከላይ አዎ ከሆነ መልስህ/ሽ መረጃውን ከየት አገኘኸው/ሽው?* | | | | 1. *ከመገናኛ ብዙሃን (ራዲዮ፤ቲሊቪዢን….)* 2. *ከስልጠናዎች* 3. *በአካባቢያችን ሰወች ሲያወሩ* 4. *ከጤና ተቁም ከባለሞያወች* 5. *ከጓደኛዬ* 6. *ከመንገድ ላይ አስተማሪዎች* 7. *ከፖስተሮች*   *88. ሌላ ካለ ይጠቀስ / /* | | | | | |  |  |  |  |  |  |  |  |
| 144 | *መልስህ መረጃ የለኝም ከሆነ ምክኒያት* | | | | 1. *መረጃውን የማገኝበት ምንም አማራጭ የለም* 2. *እኔን ስለማይመለከተኝ ፈልጌ አላውቅም* 3. *ትኩረቴን የሚስቡ ሌሎች ነገሮች ስላሉ ትኩረት አልሰጠውም*   *88. ሌላ ካለ ጥጠቀስ / /* | | | | | |  |  |  |  |  |  |  |  |
| 145 | *በአንተ እምነት ለጎዳና ልጆች ትክክለኛና በቀላሉ ማግኘት የሚቻል የመረጃ ምንጭ ይሆናል ብለህ/ሽ*  *የምታስበው/ቢው የቱን ነው?* | | | | 1. *ጓደኛን በመጠየቅ* 2. *መገናኛ ብዙሃንን (ሬዲዮ፤ ቴሌቪዢን….)* 3. *የጎዳና ላይ አስተማሪወችን* 4. *የሚበተኑ በራሪ ፅሁፎችን* 5. *ፖስተሮችን* 6. *ጋዜጦች*   *88. ሌላ ካለ ይጥቀሱ / /*  *99. አላውቅም* | | | | | |  |  |  |  |  |  |  |  |
| 146 | *ሰለ ወሲባዊና ስነ-ተዋልዶ ጤና ትምህርት ለጎዳና ልጆች በተለየ መልኩ ሲሰጥ በህይወትህ/ሽ አጋጣሚ ተሳትፈህ/ሽ ታውቃለህ/ሽ?* | | | | 1. *አዎ* 2. *አላውቅም* ***ወደ ጥ.ቁ 150*** | | | | | |  |  |  |  |  |  |  |  |
| 147 | *ለጥያቂ ቁጥር 148 አዎ ከሆነ መልስህ/ሽ ትምህርቱን ያዘጋጀው ድርጅት ማን ነበር?* | | | | 1. *መንግስት* 2. *በጎአድራጊ ግለሰቦች* 3. *በጎ አድራጊ ድርጅቶች (NGOs)* 4. *የሃማኖት ድርጅቶች*   *88. ሌላ ካለ ይጠቀስ / /* | | | | | |  |  |  |  |  |  |  |  |
| 148 | *የትምህርቱ/ስልጠናው አሰጣጥ እንዴት ነበር?* | | | | 1. *በድራማ መልክና በማዝናናት* 2. *ታዋቂ ሰዎችን በማሳተፍ ተሞክ* 3. *ጎዳናልጅ የነበረን ሰው በመጋበዝ ተሞክሮውን በማጋራት* 4. *የሚያሰለች የንግግር ትምህርት* 5. *ማስፈራሪያና ዛቻ የበዛበት* 6. *ሊላ ካለ ይጠቀስ / /* | | | | | |  |  |  |  |  |  |  |  |
| 149 | *የትምህርቱ አሰጣጥ ካልተመቸህ/ሽ በምን መልኩ ቢሰጥ ጥሩ ነው ትላለህ/ሽ?* | | | | 1. *በድራማ መልኩ* 2. *በአቻ አስተማሪወች* 3. *በታዋቂ/አርቲሰት፣እስፖረተኛ…/ሰወች* 4. *ጎዳና ልጅ በነበሩ ሰወች*   *88. ሌላ ካለ ይጠቀስ / /* | | | | | |  |  |  |  |  |  |  |  |
| - 1. ***የህይወት አመራር ክህሎትን ለማጎልበት የተደረጉ ልምምዶችን በተመለከተ*** | | | | | | | | | | |  |  |  |  |  |  |  |  |
| 150 | *በድርጅት ውስጥ ታቅፈህ/ሽ ታውቃለህ/ሽ* | | | | 1. *አወ (የድርጅቱ ስም / /* 2. *ታቅፌ አላውቅም* 3. *ታቅፌ ነበር ለቅቂያለሁ* | | | | | |  |  |  |  |  |  |  |  |
| 151 | *መልስህ/ሽ አወ ከሆነ ድርጅቱ በምን ዘርፍ ይረዳህ/ሻ ል?* | | | | 1. *በምግብ* 2. *በመጠለያ* 3. *በጤና* 4. *በትምህርት* 5. *በሞያ ስልጠና /ብረታብረት ስራ/*   *88. ሌላ ካለ ይጠቀ / /* | | | | | |  |  |  |  |  |  |  |  |
| 152 | *መልስህ/ሽ ድርጅቱን ለቅቂያለሁ ከሆነ ለምን* | | | | 1. *ሰወቹ ጥሩ አቀራረብ ስለሌላቸው* 2. *የሚሰጡት አገልግሎት ውስን ስለሆነ* 3. *የሚሰጡት አገልገሎት ፍላጎታችን ያገናዘበ ስላልሆነ* 4. *ድርጅቱ ስራ ስላቆመ*   *88. ሌላ ካለ ይተቀስ / /* | | | | | |  |  |  |  |  |  |  |  |
| 153 | *ሁኔታወች ቢመቻቹልህ/ሽ ምን መሆን ትፈልጋለህ/ሽ* | | | | 1. *ነጋዴ* 2. *ዶክተር* 3. *አስተማሪ* 4. *አውሮፕላን አብራሪ* 5. *ምንም መሆን አልችልም*   *88. ሌላ ካለ ይጠቀስ / /* | | | | | |  |  |  |  |  |  |  |  |
| 154 | *ከሚከተሉት ውስጥ የትኛውን ማድረግ ትችላለህ/ትችያለሽ (ከአንድ በላይ መልስ ይቻላል)* | | | | 1. *ኮንዶም እንዴት መጠቀም እንደሚቻል* 2. *ከፍላጎቴ ውጭ በወሲብ እዳይፈፀምብኝ ማድረግ* 3. *ከፍላጎቴ ውጭ የወሲብ ሙከራ ቢደረግብኝ ለፖሊስ ማመልከት* 4. *የወሊድ መከላከያ መቸና እንዴት መጠቀም እደሚቻል* 5. *የእግር ኳስን ጨምሮ የተለያዩ ጨዋታወችን* 6. *መኪና ማጠብና ሌሎችንም ስራ መስራት*   *88. ሌላ ካለ ይጠቀስ / /* | | | | | |  |  |  |  |  |  |  |  |
| 156 | *አብዛኛውን ጊዜህን ምን በመስራት ነው የምታሳልፈው/ፊው* | | | | 1. *በመተኛት* 2. *በመዞር* 3. *ጫት በመቃም* 4. *ብር በመለመን* 5. *ኩዋስ በመጫወት*   *88. ሌላ ካለ ይጠቀስ / /* | | | | | |  |  |  |  |  |  |  |  |
| 157 | *የጎዳና ህይወትህን/ሽን ሊቀይርልህ/ሽ የሚችል የሰለጠንክበት/ሽበት ሞያ ይኖራል* | | | | 1. *አወ (ሞያው ይጠቀስ)* 2. *ምንም የሰለጠንኩበት ሞያ የለኝም* | | | | | |  |  |  |  |  |  |  |  |
| 158 | *ከላይ መልስህ/ሽ አልስለጠንኩም ከሆነ ለምን?* | | | | 1. *ፍላጎቱ አልነበረኝም* 2. *ጥቅሙ አልገባኝም ነበር* 3. *እድሉን ማግኘት አልቻልኩም ነበር* 4. *ለስልጠናው ብር ያስፈልግ ነበር* 5. *ሌላ ካለ ይጠቀስ / /* | | | | | |  |  |  |  |  |  |  |  |
| 159 | *ባሁኑ ወቅት በምን የሞያ ዘርፍ ብትሰለጥን/ኚ ደስ ይልሃል/ሻል?* | | | | 1. *በብረታብረት ስራ* 2. *በእንጨት ስራ* 3. *በምግብ ስራ/መስተነግዶ* 4. *በሊስትሮ ሞያ*   *88. ሌላ ካለ ይጠቀስ / /* | | | | | |  |  |  |  |  |  |  |  |
| 160 | *ከመንግስታዊ ወይም መንግስታዊ ካለሆነ ድርጅት ተደረገልኝ የምትለው/የምተይው ነገር ምን አለ (ከአንድ በላይ መልስ ይቻላል)* | | | | 1. *የምግብ እርዳታ* 2. *የልብስ እርዳታ* 3. *ነፃ የኮንዶምና ሌሎች የወሊድ መከላከያ እርዳታ* 4. *ነፃ የህክምና እርዳታ* 5. *ስለ ጤናማ ወሲብና ስነ-ተዋልዶ ትምህርት* 6. *ምንም የተደረገልኝ ነገር የለም*   *88. ሌላ ካለ ይጠቀስ / /* | | | | | |  |  |  |  |  |  |  |  |
| - 1. ***የወሲባዊና ስነ-ተዋልዶ ጤና አገልግሎት አሰጣጡን በተመለከተ*** | | | | | | | | | | |  |  |  |  |  |  |  |  |
| 161 | *አደንዛዥ ዕፅን ወይም ወሲባዊ እና ሥነ-ተዋልዶዊ የሆነ ምክር ወይም ህክምና ቢያስፈልግህ/ሽ የት ነው የምትሂደው/ጅው?* | | | | | 1. *ወደ ሃይማኖት አባቶች* 2. *ወደጤና ጣቢያ* 3. *ወደቅርብ ጓደኛ* 4. *ወደ ግል ክሊኒክ* 5. *ወደ ተንቀሳቃሽ ክሊኒኮች* 6. *ወደ NGO ክሊኒኮች*   *88. ሌላ ካለ ይጠቀስ / /* | | |  | |  |  |  |  |  |  |  |  |
| 162 | *ከሌሎች በተለየ ይህን ቦታ ለምን መረጥኅ/ሽ?* | | | | | 1. *ቦታው ለሌሎች ሰወች በማያጋልጥ ሁኔታ ስለሚገኝ* 2. *ሰወቹ ችግሬን ስለሚረዱልኝ* 3. *አገልግሎቱ ቀልጣፋ ስለሆነ* 4. *ክፍያው አቅሜን ያገናዘበ ስለሆነ*   *88. ሌላ ካለ ይጠቀስ / /* | | |  | |  |  |  |  |  |  |  |  |
| 163 | *ከራስህ/ሽ ወይም ከጎደኛህ/ሽ ተሞክሮ በመነሳት ለጎዳና ልጆች ከወሲባዊና ስነ- ተዋልዶ ጋር በተያያዘ በጣም አንገብጋቢ የጤና ችግር ነው የምትለው/ይው የቱን ነው*  *(ከአንድ በላይ መልስ ይቻላል)* | | | | | 1. *ተገዶ መደፈር* 2. *በገነዘብ ተታሎ መደፈር* 3. *በምንደፈርበት ወቅት የህግ ከለላ ማጣት* 4. *በግብረስጋ ግንኙነት ለሚተላለፉ በሽታወች መጋለጥ* 5. *ላልተፈለገ እርግዝና መጋለጥ* 6. *ስለ ስነ-ተዋልዶና ወሲባዊ ጤና መረጃ ማጣት*   *88. ሌላ ካለ ይጠቀስ / /* | | |  | |  |  |  |  |  |  |  |  |
| 164 | *በምትኖርበት/ሪበት አካባቢ ለጎዳና ልጆች ኮነዶምና ሌሎች የወሊድ መቆጣጠሪያ ዘዴወችን የሚሰጥ ድርጅት ወይም የጤና ተቁዋም አለ* | | | | | 1. *አወ አለ* 2. *የለም* 3. *አላውቅም* | | |  | |  |  |  |  |  |  |  |  |
| 165 | *መልስህ/ሽ አወ ከሆነ ምን ዓይነት የጤና ተቆም ነው ያለው* | | | | | 1. *የመንግስት ሆስፒታል* 2. *የመንግስት ክሊኒክ* 3. *የግል ሆስፒታል* 4. *የግል ክሊኒክ* 5. *የቤተሰብ መምሪያ* 6. *የ NGO ክሊኒክ* 7. *የግል ፋርማሲወች*   *88. ሌላ ካለ ይጠቀስ / /* | | |  | |  |  |  |  |  |  |  |  |
| 166 | *አገልግሎቱን ለማግነት ወደ ቦታው ሂደህ/ሽ ታውቃለህ/ሽ* | | | | | 1. *አወ* 2. *ሂጀ አላውቅም* | | |  | |  |  |  |  |  |  |  |  |
| 167 | *ከላይ መልስህ/ሽ ሂጀ አላውቅም ከሆነ ለምን?* | | | | | 1. *ሰወችን ስለምፈራቸው* 2. *ዋጋውን መክፈል ስለማልችለው* 3. *ቦታው ለኔ ስለማይመች* 4. *አገልገሎቱ በሚያስፈልገኝወቅት ስለሚዘጋ* 5. *ብዙ ወረፋ ስላለ*   *88. ሌላ ካለ ይጠቀስ / /* | | |  | |  |  |  |  |  |  |  |  |
| 168 | *ወደቦታው ሂደህ/ሽ ከኆነ በዋነኛነት ምን ዓይነት አገልግሎት ነው የሚሰጡት?* | | | | | 1. *የኮነዶም ስርጭት* 2. *የወሊድ መከላከያ እንክብል ስርጭት* 3. *የእድሜ ማራዘሚያ መድሃኒት ስርጭት* 4. *የ ኤች አይ ቪ ምርመራና ምክር አገልግሎት* 5. *የቤተሰብ ምጣኔ ትምህርትና ገልግሎት*   *88. ሌላ ካለ ይጠቀስ / /* | | |  | |  |  |  |  |  |  |  |  |
| 169 | *ከሚከተሉት የወሲባዊና ስነ-ተዋልዶ ጤና አገልግሎቶች ውስጥ የትኛውን በነፃ ታገኛለህ/ሽ?* | | | | | 1. *ኮነዶም* 2. *የወሊድ መከላከያ እንክብል* 3. *ኤች አይ ቪ ምርመራና የምክር አገልግሎት* 4. *ስለቤተሰብ ምጣኔና የወሊድ መከላከያ አጠቃቀም ትምህርት* 5. *ስለ ኤች አይ ቪ መተላለፊያ መንገዶች ትምህርት* 6. *ምንም በነፃ የሚሰጥ አገልግሎት የለም*   *88. ሌላ ካለ ይጠቀስ / /* | | |  | |  |  |  |  |  |  |  |  |
| 170 | *ዛሬ አስገድዶ ወይም በገንዘብ አታሎ የመደፈር ወንጀል ቢፍፀምብህ/ሽ ምን ታደርጋለህ?(ከአንድ በላይ መልስ ይቻላል)* | | | | | 1. *ለፖሊስ አመለክታለሁ* 2. *ወደ ህክምና ቦታ እሄዳለሁ* 3. *ለጉዋደኞቸ እዲረዱኝ እነግራቸዋለሁ* 4. *ምንም አላደርግም*   *88. ሌላ ካለ ጥጠቀስ / /* | | |  | |  |  |  |  |  |  |  |  |
| 171 | *ለወሲባዊ ወይም ሥነ-ተዋልዶዊ ጤና ምክርና አገልግሎት ለማግኘት ፈልገህ/ሽ የምተሂድበት/ጅመተ ቦታ ግራ ገብቶህ/ሽ ያውቃል?* | | | | | 1. *አዎ* 2. *ከአንድም ሁለት ጊዜ* 3. *የለም*   *99. አላስታውስም /እርግጠኛ አይደለሁም* | | |  | |  |  |  |  |  |  |  |  |
| 172 | *ለጥያቄ ቁጥር 171 አዎ ከሆነ መለስህ/ሽ ምን አይነት*  *እርዳታ ነበር የፈለግሀው/ሽው?* | | | | | 1. *የኤች አይ ቪ እና ሌሎች በሽታዎች ምርመራ* 2. *የቅድማ እና ድህረ ምርመራ ምክር* 3. *አባላዘር በሽታ ህክምና* 4. *ኤች አይ ቪ በተመለከተ የእንክብካቤና እርደዳታ አገልግሎት* 5. *ኮነዶምና ሌሎች የወሊድ መከላከያ እርዳታ* 6. *ሀጋዊ ለሆነ ፅንስ ማቋረጥ* 7. *ወሊድ አገልግሎት*   *88. ሌላ ካለ ይጠቀስ / /* | | |  | |  |  |  |  |  |  |  |  |
| 173 | *ለጎዳና ልጆች የሚሰጡ የሥነ ተዋልዶ እና ወሲባዊ ጤና አገልግሎቶችን በተመለከተ እንዴት ታያቸዋለህ/ሽ?*  *(የ × ምልክት ይደረግ)* | | | | |  | | | *አወ* | | *አይደለም* | | |  |  |  |  |  |
|  |  |  |  |  |  | 1. *በጓደኝነት አቀራረብ ይሰጣል* 2. *ቆራጭ ፈላጭነት ስሜት የለባቸውም* 3. *የጓዳናን ባህል ግምት ውስጥ ባስግባ መልኩ ይሰጣሉ* 4. *አገልግሎቱ ሚስጢራዊነት የተጠበቀ ነው* 5. *ለጎዳና ልጆች በሚመች ቦታ ይገኛል* 6. *አገልግሎቱን ለማግኘት ብዙም ወረፋ የለም* 7. *ሂጀ ስለማላውቅ ምንም ማለት አልችልም* | | |  | |  | | |  |  |  |  |  |
|  |  |  |  |  |  |  |  |  |  | |  | | |  |  |  |  |  |
|  |  |  |  |  |  |  |  |  |  | |  | | |  |  |  |  |  |
|  |  |  |  |  |  |  |  |  |  | |  | | |  |  |  |  |  |
|  |  |  |  |  |  |  |  |  |  | |  | | |  |  |  |  |  |
|  |  |  |  |  |  |  |  |  |  |  | | | | |  | | |  |
|  |  |  |  |  |  |  |  |  |  |  | | | | | |  |  |  |
| 174 | *የጎዳና ልጆችን የወሲባዊና ስነ ተዋልዳዊ የጤና ችግር ለማቃለል ከሚሰረባቸው ስልቶች የትኞቹ በአነተ/ች አካባቢ ይሰራባቸዋል?* | | | | | 1. *የጎዳና ልጆችን በትምህርት በመስጠት አየተሳተፉ ነው* 2. *የመከላከል ትምህርት በበለጠ ትኩረት እየተሰጠ ነው* 3. *የወሊድ መከላከያ አቅርቦቶችን ለጎዳና ልጆች ተደራሽነት እንዲኖረው እየተደረገ ነው* 4. *ለ ኤች አይ ቪእና ላልተፈለገ እርግዝና በሚያጋልጡና በሚከላከሉ ነገሮች ላይ ትኩረት ይሰጣል*   *88. ሌላ ካለ ይጠቀስ / /*  *99. አላውቅም* | | |  | | | | | | | |  |  |
| 175 | *ለጎዳና ልጆች ወሲባዊና ስነ- ተዋልዶዊ የጤና አገልግሎትና ምክር እዳያገኙ የሚያደርጋቸው እንቅፋቶች የትኞቹ ናቸው?*  *(ከአንድ በላይ መልስ ይቻላል)* | | | | | 1. *ስለሚሰጡት አገልግሎት እውቀት አለመኖሩ* 2. *ስለ አደጋው/ችግሩ በቂ ግንዛቤ አለመኖር* 3. *መገለልንና መድሎን በመፍራት* 4. *ዋጋውን በመፍራት* 5. *ባለሙያዎች ስነ- ምግባር ችግር* 6. *የጤና ድርጅቱ መራቅ*   *88. ሌላ ካለ ይጠቀስ / /* | | |  | | | |  |  |  |  |  |  |
| 176 | *ከራስህ/ሽ ወይም ከጎደኛህ/ሽ ተሞክሮ በመነሳት ለጎዳና ልጆች በጣም አንገብጋቢ የወሲባዊና ስነ- ተዋልዶዊ የጤና አገልግሎት ችግር ነው የምትለው/ይው የቱን ነው*  *(ከአንድ በላይ መልስ ይቻላል)* | | | | | 1. *የኮንዶም ስርጭት ውስንነት* 2. *የወሊድ መከላከያ አማራጮች አቅርቦት ውስንነት* 3. *ስለ ጤናማ ወሲባዊ ህይወት ግንዛቢ እጥረት* 4. *ራስን ካልተፈለገ እርግዝና እና ከአባላዘር በሽታ ለመከላከል በቂ ግንዛቤ አለመኖር* 5. *ከወሲባዊ ጥቃት የሚጠብቀን አካል አለመኖሩ*   *88. ሌላ ካለ ይጠቀስ / /* | | |  | | | |  |  |  |  |  |  |
| 177 | *ለጎዳና ልጆች የሚሰጡ የስነ-ተዋልዶና ወሲባዊ ጤና አገልግሎቶች በምን መልኩ ቢሰጡ ውጤታማ ይሆናሉ ብለህ/ሽ ታምናለህ/ኛለሽ?* | | | | | 1. *ለጎዳና ልጆች ለብቻ በተዘጋጀ አገልግሎት መስጫ ማእከል* 2. *ባሉት የጤና ተቁዋም ውስጥ ለብቻ በተዘጋጀ ክፍል* 3. *ልጆች በሚገኙበት ቦታ ተንቀሳቃሽ አገልግሎት በመስጠት* 4. *ከጎዳና ልጆች ውስጥ በሰለጠኑ ልጆች (ባለሞያወች)*   *88. ሌላ ካለ ይጠቀስ--------------------* | | |  | | | |  |  |  |  |  |  |
| 178 | *ከፍተኛ የጎዳና ልጆችን የኮንዶም ተጠቃሚነት ለማረጋገጥ በዘርፉ የሚሰሩ ድርጅቶች በምን መልኩ ማሰራጨት አለባቸው?* | | | | | 1. *በነጻና የህዝብ ስልክ ክፍል ውስጥ በማስቀመጥ* 2. *ከግል ፋረማሲወች ጋር በመተባበር ለጎዳና ልጆች በነፃ እንዲሰጥ በማድረግ* 3. *ከጎዳና ልጆች ውስጥ በመመልመል እየዞሩ እንዲያከፋፍሉ በማድረግ* 4. *በአካባቢው በሚግኝ ቡና ቤት ውስጥ በነፃ እዲሰጥ በማድረግ* 5. *88. ሌላ ካለ ይጠቀስ* | | |  | | | |  |  |  |  |  |  |
| 179 | *አሁን ያለው አገልግሎት የጎዳና ልጆችን ፍላጎት ያረካል ብለኅ/ሽታስባለህ/ሽ?* | | | | | 1. *አወ ያረካል* 2. *በመጠኑ ያረካል* 3. *በፍፁም አያረካም* | | |  | | | |  |  |  |  |  |  |
| 180 | *ከላይ መልስህ/ሽ አያረካም ከሆነ ለምን?*  *(ከአንድ በላ መልስ ይቻላል)* | | | | | 1. *አገልግሎቶቹ በጣም አይታወቁም* 2. *የአቻ ላቻ አገልግሎት ፕሮግራም የለም* 3. *በፕሮግራም ቀረጻ፣ትግበራና ምዘና ላይ የጎዳና ልጆች ተሳትፎ የለበትም* 4. *ሚስጥራዊነት ይጎለዋል* 5. *የኮነዶም ስርጭት ችግር አለበት*   *88. ሌላ ካለ ይጠቀስ* | | |  | | | |  |  |  |  |  |  |

**አመሰግናለሁ!**

##

## Semi-structured Questionnaire

Good morning/Good afternoon! Well come to our group discussion**.** My name is __________I came from Addis Ababa University, public health school attending a post graduate study in community health department. I and my friend are here today to discuss about organizational responses for sexual and reproductive health needs of street children. You are free to talk whatever information you thought based on the topic guideline prepared. I assure you that you will not face any kind of harm for your participation in this study. Whatever information that you give me will be very useful for the study. This information will help policy makers and other organizations to design intervention activities based on research findings. I thank all of you for your willingness to participate.

Are you voluntary to participate in the study?

If yes continue

**Guidelines for focus group discussion (FGD) with street children**

| 1. Where the street children are normally found in Addis Ababa? Why? |
| --- |
| 1. Why are you coming to the stree |
| 1. What are the most common sexual or/and reproductive health problems that street children face today? |
| 1. What are the factors that agravate the above sexual and reproductive health problems? |
| 1. Are you aware of any organization which is working to help you avoid sexual and reproductive health problems you mentioned above? |
| 1. Have you ever directly participated in any HIV/ AIDS and sexual health activity organized by NGOs and Government Ministries? |
| 1. What are your views about the health education programme which address matters related to sexual and reproductive health problems for street children? |
| 1. What do street children like yourselves do when affected by the sexual health problems that you mentioned above? |
| 1. What barriers prevent you from getting information about sexual and reproductive health including HIV/AIDS? |
| 1. Do you think it is necessary for street children to participate in sexual and reproductive health and HIV / AIDS prevention education? |
| 1. Who is your source of information concerning RH, STIs & HIV/AIDS, and VCT? Do service providers and street health educators of your local inform and discuss with street children about reproductive health and sexual matters? |
| 1. Is it easy or difficult for street children in this area to find condoms or other contraceptives if they want to use them? |
| 1. Is it easy for street children to visit health facilities to obtain RH, VCT, & ART services? |
| 1. Do street children in this area visit health services when they encountered RH, STIs & HIV/AIDS problems? |
| 1. What is your recommendation regarding improvement of SRH (PMTCT , VCT , ART …) situation of street children? |

1. ***Key Informant Interview Template for service providers***

| *TO BE COMPLETED BY INTERVIEWER* | | |
| --- | --- | --- |
|  | *Name of SRH service providers* |  |
|  | *Location* |  |
|  | *Type/description* |  |
|  | *Role /position of respondent* |  |
| *S.N* | *Questions* | *Responses*  *(Record quotes and comments as well)* |
|  | *How many street children clients would you (or the service) see on average in each day/week?* |  |
|  | *What are the main sexual and reproductive health problems that street children present to your service with?* | *_________________________________________________________________________________________________________________ _____________________________________* |
|  | *What type of sexual and reproductive health services does your facility provide to street children?* | *Counseling*  *Outreach, RH services*  *Health education*  *other_________________________ _____________________________ _____________________________* |
|  | Has your organization developed, adapted or used a life-skills based education strategy for street children? | *Yes____ No _____* |
|  | *What strategies will you like to see put in place to reach street children with life skills based HIV/AIDS prevention education?* | *_______________________________________________________________________________________________________________* |
|  | *Are there any sexual and reproductive health services that you are unable to provide to street children but you think you should be providing?* | *Yes____ No _____* |
|  | *If yes, please specify?* | *_____________________________________ _____________________________________ _____________________________________ _____________________________________ _____________________________________ _____________________________________* |
|  | *What are the main barriers/problems that service providers face in providing sexual and reproductive health services to street children?* | *policy restrictions*  *lack of res*  *lack of training etc*  *other__________________________ _______________________________ _________________________________* |
|  | *Have you taken any steps to make street children comfortable using the Services and to create a “street children -friendly “environment?* | *Yes____ No_____* |
|  | *If yes, please describe* | *_____________________________________ _____________________________________ _____________________________________ _____________________________________ _____________________________________ ______________________________________* |
|  | *Do you work in collaboration with any other organizations in providing sexual and reproductive health services for street children?* | *Yes______ No ______* |
|  | *if yes ,please specify your collaborative organizations* | *______________________________________ ______________________________________ ______________________________________ ______________________________________* |
|  | *How do you promote your sexual and reproductive health services to street children?* | *______________________________________ ______________________________________ ______________________________________ ______________________________________ ______________________________________* |
|  | *Generally, how comfortable would you say you feel in providing sexual and reproductive health services to street children?* | *______________________________________ ______________________________________ ______________________________________ ______________________________________ ______________________________________ ______________________________________* |
|  | *Do you think is good about the services provided by you to Street children? Why?* | *______________________________________ ______________________________________ ______________________________________* |
|  | *When consulting with street children about sexual and reproductive health, what steps do staff members take to ensure privacy/confidentiality?* | *__________________________________________________________________________ ______________________________________ _________________________________________________________________________* |
|  | *How confident do you feel staff members are in providing SRH services for street children?* | *____________________________________________________________________________* |
|  | *Have staff members received any training in street children health?* | *Yes______ No ______* |
|  | *If yes, please describe any training received* | *______________________________________ ______________________________________ ______________________________________* |
|  | *In which topics/skills do you think that staff members need training in order to provide effective sexual and reproductive health services to street children?* | *___________________________________________________________________________ __________________________________________________________________________* |
|  | *Are former street children hired and trained as service provider in your service delivery activity to ensure friendly service?* | *Yes_________ No___________* |
|  | *Are there any practice guidelines/medical literatures available to guide staff in providing preventive and curative services for street children sexual and reproductive health problems?* | *Yes_________ No _________* |
|  | *Please list things that you think should be done to improve the quality of sexual and reproductive health service provision to street children?* | *________________________________________________________________________________________________________________________________________________________________________________________________________________________________* |
|  | *Do staff members have sufficient resources/equipment to effectively provide services to street children? (E.g. clinical supplies; condoms; IEC materials, etc)* | *Yes_______ No___________* |
|  | *How do you reach them with your activities? Please name the activities?* | *_____________________________________ ___________________________________________________________________________* |
|  | *Is it possible for street children to drop in and receive sexual and reproductive health services without an appointment when they are in need?* | *yes__________ No___________* |
|  | *If no, how long on average do street children have to wait before receiving sexual health services?* |  |
|  | *Does your service charge any fees for services to street children?* | *yes___________ No____________* |
|  | *If yes, which services and how much?* | *________________________________________________________________________________________________________________* |
|  | *Are there any groups of street children for whom you feel the service does not Cater well?* | *Yes________ No__________* |
|  | *if yes ,list them* | *_____________________________________* |
|  | *Are there any challenges or successes in your activities you may want to share with us?* | *____________________________________________________________________________ ____________________________________________________________________________* |
|  | *if you want to say something related to sexual and reproductive health services in your facilities :* | *____________________________________________________________________________ __________________________________________________________________________________________________________________* |
